# R5 Peptides Constitute Condensed Phases with Liquid-Like Properties in Biomimetic Silica Capsules

**DOI:** 10.1021/acs.jpclett.5c00144

**Published:** 2025-04-23

**Authors:** Dörte Brandis, Giulia Mollica, Dennis Kurzbach

**Affiliations:** † Institute of Biological Chemistry, Faculty of Chemistry, University of Vienna, Währinger Str. 38, 1090 Vienna, Austria; ‡ University of Vienna, Vienna Doctoral School in Chemistry (DoSChem), Währinger Str. 42, 1090 Vienna, Austria; § Aix Marseille Univ, CNRS, ICR, 13397 Marseille, France

## Abstract

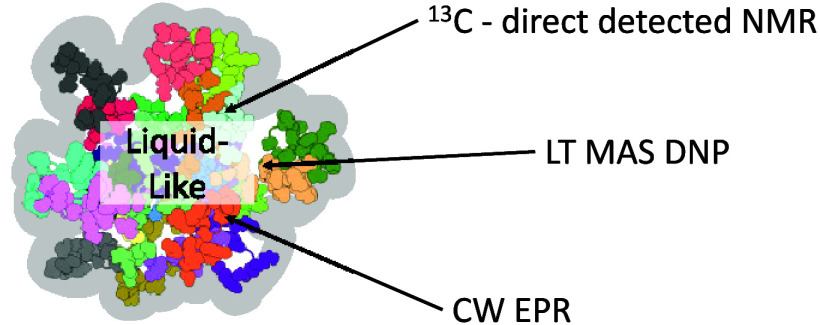

Biomimetic silica-peptide nanocomposites are promising materials for applications in drug delivery and enzyme encapsulation due to their biocompatibility, tunable morphologies, and unique structural characteristics. However, the structural dynamics of the peptide scaffold remain largely elusive, impeding rational biomimetic materials design. This shortcoming is not the least due to a lack of methods that can access such heterogeneous systems with dynamics on a wide range of time scales. Among the most studied candidates are silica particles templated by the diatom-derived peptide R5, known for its ability to guide silica precipitation under mild, toxicologically friendly conditions, leading to silica capsules filled with a peptide scaffold. Here, we describe the structural dynamics of R5 within its self-assemblies and the silica particles it templates with a combination of advanced magnetic resonance methods, including ^13^C-direct detected NMR, site-directive spin-labeling EPR, and sensitivity-enhanced solid-state NMR. We provide evidence that R5 self-assemblies form condensed phases with liquid-like dynamics both before and after silica encapsulation. Our suite of methods allowed us to access R5/silica composites over a comprehensive range of time scales. These results demonstrate that R5 retains a remarkable degree of internal dynamics, with distinct regions of solid-like and liquid-like behavior even within the silica particles. Specifically, the peptide scaffold comprises three dynamic species: (i) solid-like at the peptide-silica interface, (ii) liquid-like mobility within the scaffold core, and (iii) intermediate dynamics at the boundary regions between core and interface species. Our findings rationalize the high mobility of guest molecules, such as drugs or enzyme substrates, within R5-silica nanoparticles, which is crucial for their functionality in controlled release and catalytic applications. This understanding paves the way for improved rational design considerations for advanced nanomaterials and expands our knowledge of biomimetic mineralization mechanisms. At the same time, the methodological approach can be useful for many types of peptide-guided biominerals, bridging fundamental biochemistry with biotechnological innovation.

Biomimetic silica nanoparticles provide great potential in applications from drug transport to enzyme encapsulation due to their biocompatibility, low toxicity, availability, tunable density/porosity, and surface properties.^1−6^ In this context, one of the most prominent and biotechnologically important routes toward tailoring silica particles is based on diatom-derived peptides, such as the 19 amino acid-long R5.^7−10^

The R5 peptide, derived from the diatom *Cylindrotheca fusiformis*,^11−13^ has emerged as a major platform in biomimetic materials science. Its significance lies in its ability to self-assemble and, thereby, adopt a catalytic role in the formation of functional silica nanoparticles under mild conditions, which mimic natural biomineralization processes.^14−16^ The R5 peptide’s functionality is currently extensively studied for applications in bioinspired nanomaterials,^17^ drug delivery,^18^ and biosensors,^19^ aiming to exploit its environmentally benign synthesis pathway and capability to produce highly defined silica particles.^20^

Traditional silica formation methods, such as sol-gel chemistry, often require extreme pH conditions, organic solvents, or high temperatures, which can compromise the stability and functionality of biological molecules. In contrast, nature-inspired approaches utilizing peptides, such as R5, enable the controlled deposition of silica under aqueous conditions at neutral pH.^7−9,16,21^ It facilitates silica formation by promoting biomimetic mineralization, leading to tunable porosity, enhanced structural stability, and the preservation of biocompatibility.^8,14,21^

Recently, it was shown that the silica-precipitating activity of R5 is influenced by its sequence and structural properties, with the C-terminal region, particularly an RRIL motif, playing a critical role in particle morphology and assembly.^7,22^ Despite its wide adoption in biomaterials research, the exact mechanistic underpinnings of R5-mediated silica formation remain a topic of active investigation, with ongoing studies employing tools such as NMR spectroscopy and molecular dynamics simulations to unravel its structure-function relationships.^23−29^

Currently, it is well-established that the self-assemblies form at low millimolar concentrations in the presence of counterions such as phosphates via salt bridges involving said RRIL as well as an N-terminal SKKS motif.^24^ These templates can recruit silicic acid from solution at moderate millimolar concentrations and condense it into highly defined, monodisperse particles with tailored morphologies (see Figure 1 for a sketch of the process).^30^ Biotechnologically, most importantly, the resulting particles can be loaded with enzymes, drugs, or other cargo to enable controlled release applications or create nanoreactors. Such applications are particularly efficient in the case of R5 due to the high mobility of internalized compounds as well as efficient diffusion through their porous silica shells.^31^ These features prevail even despite the high stability and rigidity of the silica.^16,18^

**Figure 1:**
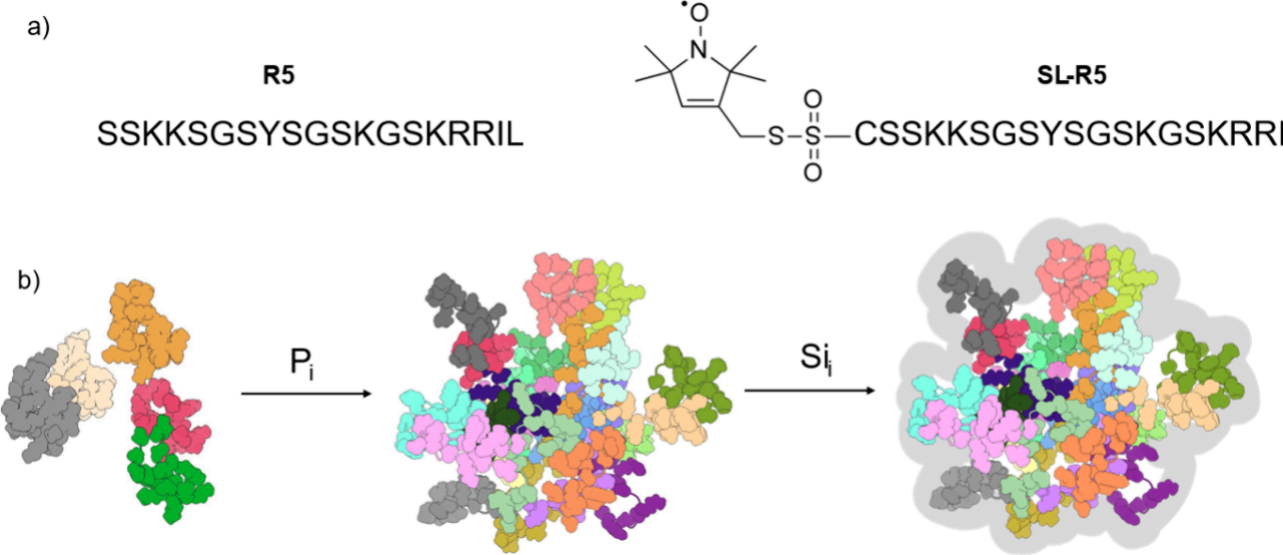
a) Structure of R5 and its variant with a spin-labeled N-terminal cysteine. b) Schematic representation of silica precipitation process. The R5 peptides are homogeneously dissolved in water before the addition of phosphate counterions, which induces self-assembly. The subsequent addition of silicic acid leads to the formation of a silica shell around these assemblies (simplified representation).

Understanding the molecular configuration of R5-based particles is, naturally, a crucial step in their rational design for application development. To achieve this, significant efforts have been made to decipher the structure of the silica shell, its size, and its porosity, as well as to understand the interplay between the silica shell and the internal peptide scaffold.^15,24^ The morphology of the silica particles was, *e.g*., just recently shown to be directly determined by the shape of R5 self-assemblies that become silica-coated in several consecutive steps.^20,32^

However, despite the intense ongoing research efforts, the internal structural dynamics of R5 self-assemblies before and after silica coating remain largely unknown. This is a critical shortcoming as the biotechnological use of R5 depends heavily on the cargo-accessibility of the particle’s inner volume as well as on the mobility of guest molecules within. This crucial lack of knowledge is not the least due to the limitation of available analytical methods to characterize such systems that feature heterogeneous structural dynamics on several length and time scales.

To overcome this hurdle and address this knowledge gap, we herein suggest a multimodal approach integrating several advanced spectroscopic techniques. These included ^13^C-direct detected nuclear magnetic resonance (NMR)^33,34^ and electron paramagnetic resonance (EPR) in combination with site-directed spin labeling (SDSL), as well as low-temperature magic angle spinning dynamic nuclear polarization (LT MAS DNP).^35,36^ In combination, these tools allowed us to shed some light on the internal dynamics of R5 self-assemblies before and after silica encapsulation.^37^

With our approach, we reveal that R5 self-assemblies form condensed phases with liquid-like internal dynamics reminiscent to some degree of liquid-liquid phase separation phenomena.^38−40^

Remarkably, the liquid-like dynamics are partially retained even after silica encapsulation, explaining the high mobility of guest molecules and the accessibility of the nanoparticles’ interior. These observations not only rationalize the advantageous properties of R5-based silica nanoparticles but also provide new insights into the fundamental biophysical mechanisms underlying peptide-templated silica formation. By advancing our understanding of these processes, this work can help foster more informed design considerations for next-generation biomimetic nanomaterials with tailored functionalities.

Upon exposure to millimolar phosphate (P_i_) concentrations in slightly acidic aqueous buffers (herein *c*(P_i_) = 50 mM, pH = 6.5), R5 self-assembles into large aggregates with molecular weights surpassing 1 MDa in many cases.^20^ The morphologies of the resulting suspended species are highly sensitive to phosphate concentration, the phosphate-to-R5 molar ratio, and the overall solution conditions.^9,20,23^ Recent studies have demonstrated that the self-assembled structures of R5 play a decisive role in dictating the shape of the silica particles for a wide range of conditions.^20^ Indeed, the morphology of the silica particles directly reflects the underlying self-assembly scaffold, with the silica coating adopting the shape of the peptide assemblies (Figure 1b). These findings highlight the critical interplay between the structural properties of the peptide-based template and the final silica nanoparticle architecture. To understand the structural dynamics of the peptide scaffold and its implications in hosting guest molecules, we first investigated the suspended self-assemblies and then the final silica particles.

We studied the internal dynamics of R5 in its self-assembly before silica coating via ^13^C′-detected ^15^N-*R*_1_ and ^15^N-*R*_2_ NMR relaxation rate constants.^41,42^ Our earlier work showed that the typically ^1^H-detected ^15^N-relaxation rate constants only reflect minor populations of monomers remaining in solution outside the self-assemblies as proton-exchange processes within the assemblies cause the corresponding resonances to be broadened beyond detection. This issue is effectively canceled using the suggested heteronuclear detection scheme.^20^

This feature is demonstrated in detail in our earlier work^20^ and additionally visualized by the ^13^C-^15^N correlation spectrum in Figure 2a. Upon self-assembly, the R5 chemical shifts are significantly perturbed, while the signal intensities are not drastically reduced. The former reports a changing environment upon self-assembly, and the latter indicates that the majority of R5 resonances remain detectable. Hence, the ^13^C-direct detected correlation spectra efficiently pick up the resonances of R5 within the self-assemblies.

**Figure 2:**
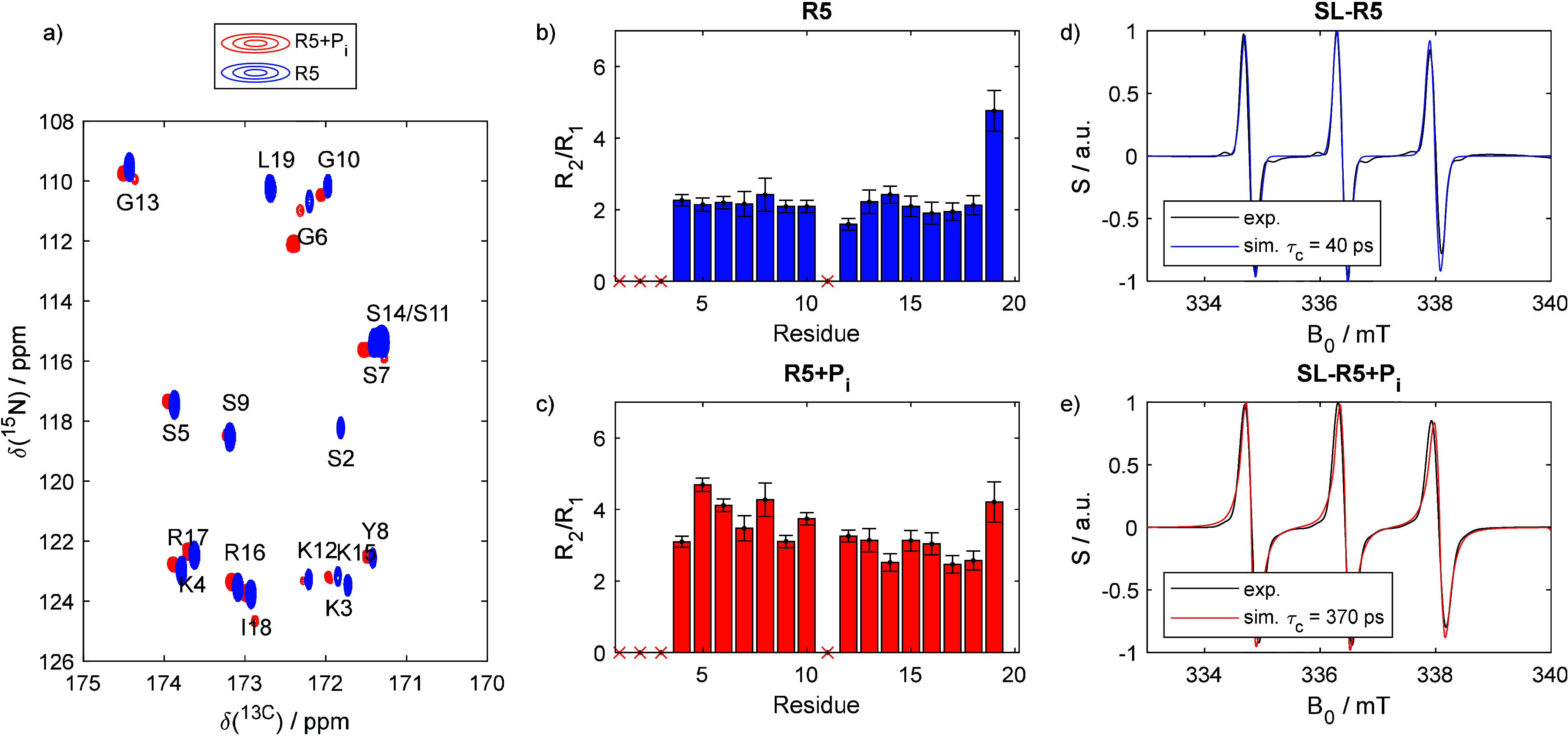
a) Liquid-state ^13^C-^15^N correlation spectrum of R5 before (blue) and after (orange) phosphate-induced self-assembly. The arrow indicates how residue L19 shifts upon exposure to P_i_. b) ^15^N-*R*_2_/*R*_1_ ratios for R5 in phosphate-free solution. Note that residues marked with red crosses could not be analyzed due to weak signal intensities. c) ^15^N-*R*_2_/*R*_1_ ratios for R5 in a 50 mM P_i_-containing buffer. d) CW EPR of free SL-R5 (black) and the corresponding spectral simulation (blue). e) CW EPR of self-assembled SL-R5 (black) and the corresponding spectral simulation (red).

It should be noted that the peptide resonances stem from an exchange average between free and assembled forms. Considering that the amount of free R5 in solution is <1%,^20^ as determined in our earlier work under similar conditions, they nonetheless report almost exclusively on the assembled species.

With the working detection scheme in hand, we moved on to investigate the ^15^N relaxation rates before and after self-assembly. The effect is efficiently visualized through residue-resolved *R*_2_/*R*_1_ ratios (Figure 2b,c), which reflect the rotational mobility of the individual amino acids. (All related *R*_1_ and *R*_2_ data can be found in Supporting Information Figures S1–S6.) Under the assumption of simple spherical rotation, these ratios would correspond to the correlation time τ_c_. However, as R5 features more complex conformational dynamics, the *R*_2_/*R*_1_ ratios do not represent correlation times directly but instead reflect a qualitative, relative measure for residue mobility, *e.g*., between states before and after self-assembly.

The free peptide in solution displayed *R*_2_/*R*_1_ values of ca. 2 (apart from the N- and C-terminal regions). In the presence of 50 mM P_i_, values up to 5 were detected. This change shows that the different residues become dynamically more restricted upon self-assembly,^43^ which can be expected due to the salt bridges between the R5 peptides and as the environment becomes very crowded.

Notably, the size of the assemblies is on the order of hundreds of nanometers, for which one would expect much higher *R*_2_/*R*_1_ ratios ≫10, if the particles were mere solids.^43^ To rationalize this discrepancy, it has to be assumed that the internal dynamics of R5 within the self-assemblies are still relatively fast, much like in condensed liquid phases of intrinsically disordered proteins.^44−47^ This observation aligns well with earlier reports that also indirectly pointed toward high residual mobility.^20^

The *R*_2_/*R*_1_-derived conclusion was then further substantiated by EPR spectroscopy (Figure 2d,e), which assessed rotational correlation times directly. We engineered an R5 variant with an N-terminal cysteine, a modification previously shown to preserve the full functional profile of unmodified R5.^21^ The cysteine was subsequently labeled with the nitroxide spin label MTSL (S-(1-oxyl-2,2,5,5-tetramethyl-2,5-dihydro-1H-pyrrol-3-yl)methylmethanesulfonothioate; see Figure 1a). The spin-labeled R5 (SL-R5) variant was then analyzed using continuous wave (CW) EPR spectroscopy both before and after self-assembly. CW EPR is particularly sensitive to changes in the rotational motion of the spin label, as reflected in the line shape of the unpaired electron’s resonances.

Spectral simulations of the CW EPR line shapes (Figure 2d,e) revealed a reduction in spin-label mobility upon self-assembly. Specifically, the rotational correlation time (τ_c_) of the spin label increased from 0.04 to 0.37 ns. At the same time, the Voigtian line width broadened from 5.9 to 6.4 MHz, both of which are indicative of reduced motional freedom following self-assembly (simulation parameters are provided in Supporting Information Table S1). Importantly, however, the EPR spectra retained a solution-like character even after self-assembly, as evidenced by the overall line shape and the still relatively short τ_c_.

These findings confirm that SL-R5 undergoes efficient self-assembly, leading to restricted local mobility. Nevertheless, the preserved solution-like dynamics within the assemblies, reflected in the EPR line shape and the rotational correlation times, highlight the retention of fast dynamics within the self-assembled state.

Together, the EPR and NMR data clearly show that R5 self-assembles into condensed phases with internal mobility that retains liquid-like dynamics to some degree. In the next step, we investigated whether this peculiar condition is retained upon silica coating of the self-assembly surfaces.

Note that we chose the notion of “condensed phases with liquid-like dynamics” not to imply any specific relations to liquid-liquid phase separation,^38,48^ peptide condensation^20,32^ or similar phenomena^49^ that recently received ample attention and are subject to particular definitions in terms of phase behavior. Instead, we simply want to refer to a liquid-like character in terms of molecular dynamics of peptide-rich R5 phases while not implying any specific definition.

To explore the structural dynamics within the silica self-assemblies, we again employed SL-R5 in combination with CW-EPR.

Using established protocols,^20^ we coated the R5 assemblies with silicic acid, forming silica nanoparticles. Figure 3 shows the corresponding SEM (scanning electron microscopy) micrographs. These nanoparticles were then subjected to multiple wash cycles.

**Figure 3:**
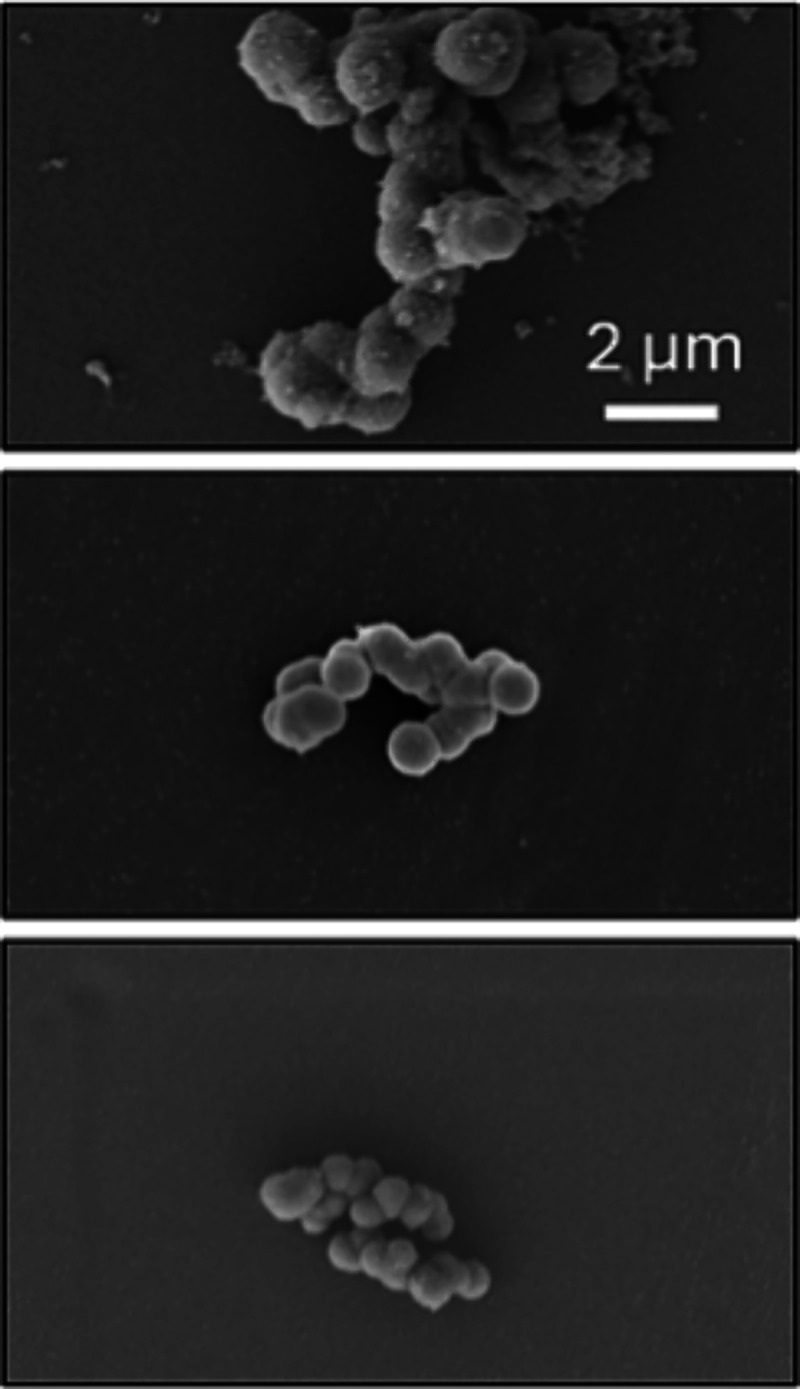
SEM images of the nanoparticles formed by SL-R5 upon exposure to silicic acid.

These cycles were important as they ensured that no R5 remained outside of the silica particles. By determining the peptide concentration via EPR, NMR, and UV absorption in the supernatant in every wash cycle, we ensured that the R5 concentrations in the surrounding buffers remained below the detection threshold for every experiment (see Supporting Information Figures S7 and S8).

The resulting R5/silica composites were then analyzed using CW EPR spectroscopy at room temperature. In contrast to solution-state NMR, SDSL-EPR with nitroxides offers the advantage of being able to detect signals across a broad range of molecular motions, simultaneous from the fast- to the slow-tumbling and the solid regime.^50,51^

This feature proved necessary for investigating SL-R5 within the silica capsules, as it allowed us to reveal several species with drastically different conformational dynamics.

X-band EPR spectroscopy is sensitive to molecular dynamics with rotational correlation times ranging from 10 ps to ca. 50 ns,^52,53^ capturing motion on time scales from fast tumbling to near-rigid behavior. In terms of concentration, CW-EPR can detect radicals down to 10^–9^ M. At cryogenic temperatures (∼4 K), sensitivity improves, allowing detection limits as low as 10^–10^ M under ideal conditions and especially in systems with long *T*_1_ times.^52,53^ In comparison, higher-frequency EPR techniques (e.g., Q-band at 35 GHz or W-band at 95 GHz) provide enhanced resolution but often demand higher sample concentrations.

Figure 4a displays the spectral components used to model the SL-R5 CW EPR spectrum within the silica capsules: a sharp liquid-state species, a broad solid-state species, and an additional heavily broadened liquid-state species. Figure 4b shows combinations of either all three or only the narrow liquid and solid species and compares them to the experimental spectrum. These comparisons show that the third species significantly improves the match, leading to good agreement between the simulated and experimental spectrum. Using only the fast and the slow components, no satisfactory match could be achieved. The simulation parameters are listed in Table 1 and the Supporting Information Table S2.

**Figure 4:**
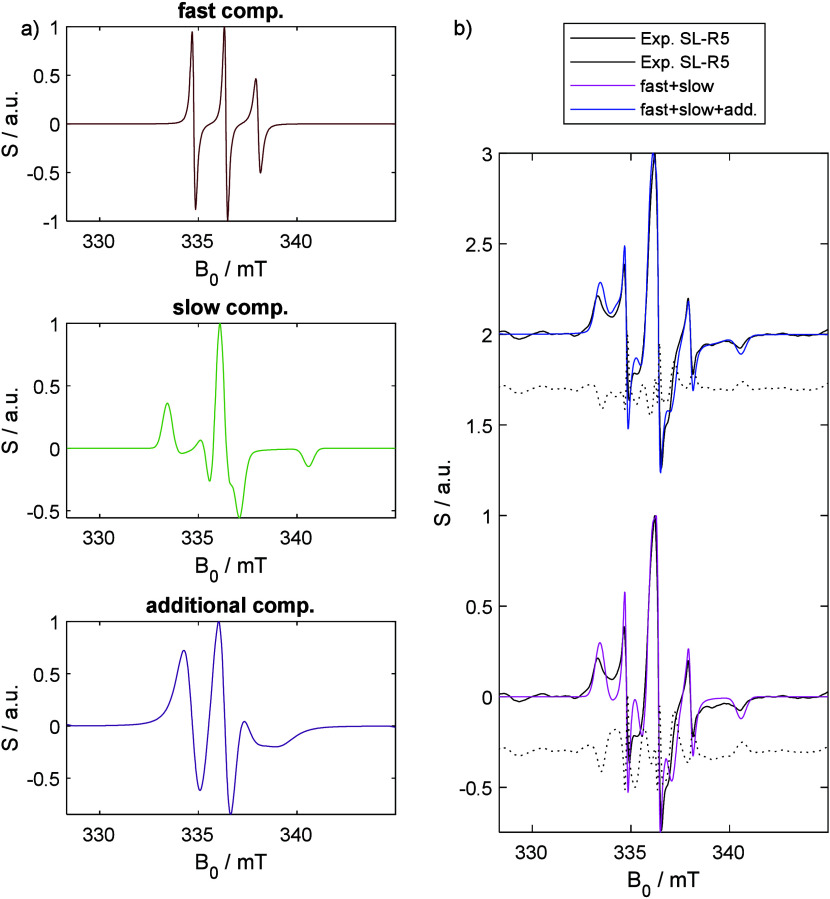
a) Simulated components of the CW EPR spectrum of SL-R5 encapsulated in silica shells. b) Combinations of either all three (blue) or only the liquid and solid species (magenta) of panel (a) and the experimental CW EPR spectrum (black). Residuals are shown as dotted lines.

**Table 1:** Key Parameters Used for Simulations of the EPR Spectrum of SL-R5 Containing Silica Particles^a^

**Component**	**fast**	**slow**	**additional**
*a*_iso_/A-tensor/MHz	42.3	[18, 18, 104]	[18, 18, 109]
Log_10_(*D*_rot_)/rad^2^ ns^–1^ b	[7.5, 9.0, 8.0]	n/a	[8.0, 8.0, 8.5]
Exchange frequency/ms^–1^ b	40	n/a	10
Weight fraction^b^	0.4	0.5	0.1

a Tensors are given as principal components in the order [xx, yy, zz]. All other simulation parameters can be found in Supporting Information Table S2.

b The reported exchange frequencies and relative populations are the values fed into our spectral simulations to match the experimental data. However, given the complexity of EPR spectra in the slow-motion regime, including anisotropic rotational diffusion, it cannot be ruled out that other combinations of parameters would lead to similar results. Therefore, it is important to note that our data interpretation does not rely on quantification of exchange frequencies of relative populations, but on the mere observation that only two components (fast and slow rotating species) could not provide a good match to the experimental data.

The presence of the solid-state component is expected, consistent with previous reports of R5’s N-terminal region binding directly to the silica capsules’ inner surface.^24^ However, the detection of a fast-tumbling species is notable as this directly proves liquid-like dynamics to prevail within the silica capsules. Similarly, the presence of the third species, which is heavily broadened, again points toward rapid internal dynamics within a dense network of peptides—most likely at the interface between regions of fast motion and solidified R5.

This finding indicates that the mobility of peptides within the condensed assemblies, which form in solution before silica coating, is preserved post solidification for a large share of incorporated R5. Therefore, these particles can be conceptualized as solid silica/peptide shells encapsulating a core of condensed peptides with liquid-like dynamics. Importantly, compared to SL-R5 before encapsulation, all species yet show much slower rotational movement.

Further, this interpretation is supported by the isotropic hyperfine coupling constant *a*_iso_ to the ^14^N nucleus of the spin label, reducing from 46 MHz in water to 42.3 MHz upon enclosure in the capsules.^54^ A drop in *a*_iso_ corresponds to an increase in hydrophobicity of the spin labels environment.^54^ In other words, the reduced value indicates peptide-rich surroundings as opposed to a primarily aqueous environment experienced before self-assembly.

Furthermore, successful spectral simulation of the liquid-state EPR component required accounting for Heisenberg-type exchange interactions (*i.e*., repeated spatial encounters between different SL)^50^ for both fast-tumbling species, implying that the MTSL labels are in close proximity and engage in dynamic interactions with other labels inside the particles. This suggests a high degree of internal translational mobility within the peptide scaffold.

Finally, we confirmed the liquid-like behavior observed by CW EPR within the R5-silica composites by LT MAS DNP—a method to boost the sensitivity of NMR signals through crosstalk with stable radicals, often denoted as polarizing agents (PA).^55^ Capitalizing on the high porosity of the silica shell^21^ and assuming fast internal dynamics of the R5 matrix within the particles, we conceived an experiment in the fashion of a guest-molecule loading application, much like in drug delivery; see, *e.g*., ref (56). In this experiment, a polarizing agent (instead of a cargo molecule) diffuses into the silica particles to then allow for signal-enhanced NMR studies of the capsules inside. Such an experiment would not be possible if the R5 scaffold within did not allow for efficient diffusion, as PA could not approach the peptides (Figure 5a).

**Figure 5:**
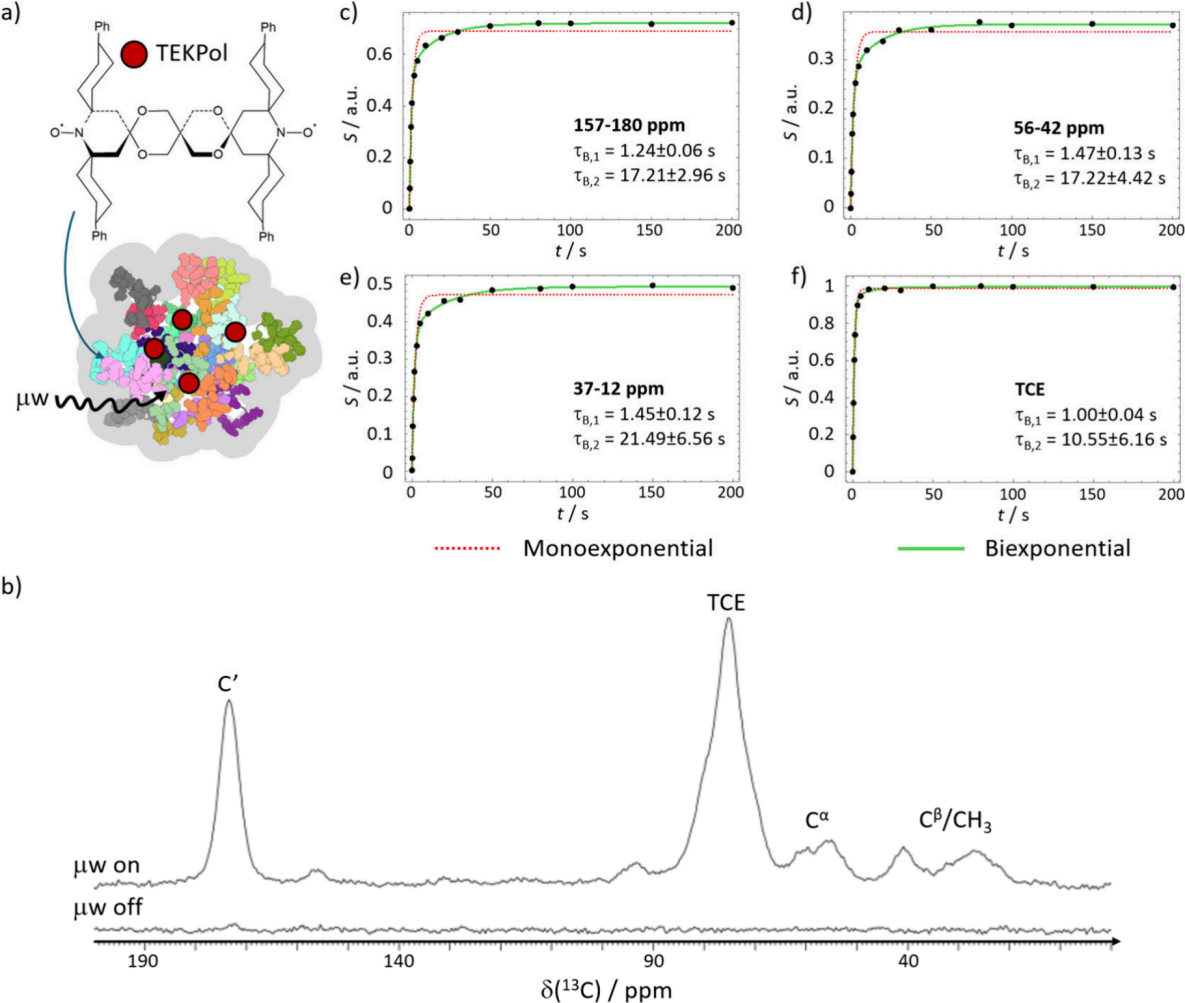
a) Scheme of LT MAS DNP experiments. The PA TEKPol diffuses into the silica-R5 composites and enhances solid-state NMR signals upon microwave irradiation. b) ^13^C LT CPMAS DNP NMR spectra of R5 within silica capsules at natural abundance. Top: under microwave irradiation. Bottom: without microwave irradiation. ^13^C build-up curves (saturation recovery) of c) carbonylic, d) alpha and e) beta and methyl carbon sites of R5 and f) TCE acquired under microwave irradiation.

Further, the silica shell is typically much thicker than the internal peptide scaffold, *i.e*., on the order of several hundreds of nanometers.^20^ This distance is much longer than the spin diffusion length under our MAS-DNP conditions.^57^ Hence, the hyperpolarization could not reach the peptide scaffold if all PAs remained outside the silica particles.

We impregnated (at room temperature) the composites with a 15 mM solution of the PA TEKPol^58^ in TCE (tetra-chloro ethane) and let it diffuse into the particles. The resulting TEKPol-loaded particles were then submitted to a MAS DNP-NMR spectrometer operating at a magnetic field of *B*_0,DNP_ = 9.4 T and at a temperature of *T*_DNP_ = 100 K. Upon microwave irradiation, the polarizing agent led to efficient signal enhancements of the ^13^C NMR resonances of the enclosed peptides (Figure 5b) via cross-polarization from nearby ^1^H nuclei. Contrastingly, only a very small solid-state NMR signal could be detected in the absence of microwave irradiation for the same number of scans, which led to a tentative estimation of ε_^13^C_ ≥ 70 for the peptide CO signal (the only signal that could be detected under μw-off conditions).

The DNP enhancement observed for R5 within the silica particles provides clear evidence for the PA’s close proximity to the peptides. This proximity, in turn, reflects the particles’ high internal mobility at room temperature, facilitating the efficient diffusion of guest molecules before sample freezing.

Interestingly, the hyperpolarization build-up curves for the R5 ^13^C′, ^13^C^α^, ^13^C^β^ and ^13^CH_3_ resonances (Figure 5c–e) exhibited biexponential behavior. In contrast, the build-up function for TCE, used to deliver the TEKPol radical into the particles, followed a monoexponential trend consistent with the behavior of a homogeneously dispersed molecule (Figure 5f).^59^ The build-up time for TCE coincides with the build-up time of the shortest component for all the investigated R5 carbon sites, again suggesting that a fraction of R5 is in close contact with the polarizing agent solution. As the kinetics of the DNP build-up depends strongly on the local concentration of PAs,^60,61^ the biexponential nature of the curves points toward the presence of at least two distinct R5 phases within the silica capsules, each with different exposure to the TEKPol solution and, consequently, different DNP efficiencies.

This finding aligns well with the EPR-derived evidence for the coexistence of solid-like and liquid-like R5 phases. The two phases likely represent regions with varying peptide density and dynamics, further underscoring the heterogeneous internal environment within the silica particles.

Finally, it should be noted that rotational diffusion plays no role in the interpretation of the MAS DNP spectra, as these were carried out at a temperature of 100 K. Therefore, a direct comparison of the EPR and the DNP data in terms of relative contributions of the different phases is not possible in a straightforward manner. However, the presence of two well distinct build-up time components strongly supports the presence of at least two (vitrified) R5 phases with different radical concentrations, hence, agreeing with the identification of multiple dynamic components by EPR.

The structural dynamics of silaffins and their derivatives within the nanostructures they template are key parameters for determining the functional accessibility of the particles’ interiors. This feature is central for host-guest interactions in drug delivery applications and the use of encapsulated enzymes and nanoreactors. Our study provides a methodology for shedding light on the dynamics of the biotechnologically crucial silaffin-derivative R5. It provides experimental evidence for the presence of a condensed liquid phase within these capsules. Our combined ^13^C-detected NMR, SDSL-EPR, and LT MAS DNP data show that the internal dynamics of R5 self-assemblies before and after silica coating show domains characterized by different dynamic behaviors, including liquid-like domains.

This finding has two important implications. (*i*) From a biotechnological viewpoint, it explains well why diatom-peptide-based silica particles have excellent properties for enzyme encapsulation and drug transport applications. Particularly, we demonstrate that the interior contains at least three different dynamic species, which constitute different parts of the R5 scaffold, from the interface with the silica shell with solid-like dynamics to the core peptides with liquid-like properties. (*ii*) From the viewpoint of fundamental biochemistry, our findings point toward the implication of condensed phases of proteins and peptides in compartmentalization and the formation of biominerals such as the cell walls of diatoms. Furthermore, due to the well-defined spherical morphology of the silica capsules, they provide a platform for studying the implications of condensed phases with liquid-like properties *in vitro*. As the relationship between structural dynamics and activity in such phases remains largely unclear, their rationalization might help to advance the design of biologically inspired nanomaterials.

## Methods

### Sample Production

The R5 peptide was either subcloned as a His-tagged SUMO-fusion construct containing an additional N-terminal Cysteine (C-R5) or as a His-tagged SUMO-fusion construct containing a TEV-cleavage site leading to an additional N-terminal Glycine (G-R5) into a pET-21a(+) expression vector and transformed into *E. coli* Rosetta 2 cells. For protein expression, the bacteria were grown at 37 °C in 2YT media before being transferred to M9 medium for ^13^C and ^15^N labeling, achieved by supplementing the medium with ^13^C_6_-glucose and ^15^N-ammonium chloride at a concentration of 1 g/L. For the unlabeled C-R5 variant, the transfer to minimal medium was skipped, and the expression was continued in 2YT-medium. When the optical density at 600 nm (A600) reached 0.6, cells were induced with isopropyl-β-d-thiogalactopyranoside and incubated overnight at 30 °C.

The harvested cells were homogenized in a buffer containing 25 mM TRIS, 100 mM NaCl, and 2 mM β-mercaptoethanol at pH 8; for the C-R5, the buffer was additionally supplemented with 2 mM DTT. The resulting supernatant was purified via Ni^2+^-affinity chromatography and the fractions were pooled upon detection of masses of 17 175 kDa (^15^N-^13^C-labeled G-R5) and 15514 kDa (C-R5) in LC-MS analyses. His- and SUMO-tags were cleaved using SUMO protease digestion overnight for C-R5 and Tev Protease for G-R5, with mass spectrometry confirming masses of 15.007 kDa for His-SUMO-tev (^15^N-^13^C-labeled) and 13415 kDa for His-SUMO (unlabeled).

The cleaved peptide mixture was further purified using a Kromasil C4 semipreparative RP-HPLC column on a Waters Prep 150 System. A gradient of 5%–65% water/acetonitrile (0.08% v/v in water/trifluoroacetic acid, 0.01% v/v) was applied over 30 min at a flow rate of 5 mL/min. Fractions with UV absorption exceeding 50 mAU were automatically collected. For analysis, 15 μL of each fraction was directly injected into a Thermo Fisher HPLC-MS system to identify product-containing fractions, which were pooled accordingly and lyophilized (Supporting Information Figure S9). For all experiments, the final peptide concentration was adjusted to 2 mg/mL (in the bulk before silica precipitation).

For SL-R5, a similar procedure was followed, with an additional MTSL labeling step achieved by incubating the peptide with MTSL in 25 mM Tris-buffer pH = 8, overnight at room temperature. Labeling efficiency was confirmed by mass spectrometry, reporting a mass of 2.3 kDa. Excess MTSL was removed using RP-HPLC. The labeling efficiency determined by mass spectrometry was ∼100%; see Figure S9.

### NMR

All 2D NMR spectra were acquired using a Bruker NEO 600 MHz spectrometer equipped with a cryogenically cooled Prodigy TCI probe. Carbon-detected ^15^N relaxation rates were recorded with varying delays as detailed in the Supporting Information and the pulse programs as detailed in refs (34, 42, and 62). Spectral widths and offsets were 4545.450 Hz/174.000 ppm (^13^C) and 1824.688 Hz/122.0 ppm (^15^N). Data processing was conducted using TopSpin 4.2 and analyzed with CcpNmr Analysis 2.5.2. Data were zero-filled to four times the number of points and apodized using a shifted sine-bell squared window function before Fourier transformation. Baseline correction was applied using a fifth-degree polynomial function. Finally, all data were fitted to monoexponential decay functions to extract the relaxation rate constants.

### CW EPR

EPR spectra were recorded at room temperature using a Bruker ESR5000 benchtop CW EPR spectrometer operating at a microwave frequency of 9.4 GHz. The precise magnetic field/microwave frequency was readjusted/determined before every experiment using the internal routine of the ESR5000. The modulation amplitude and sampling frequency were 0.2 mT and 0.16 mT/s for solution-state spectra and 0.33 mT/s for solid-state spectra. The modulation amplitude was chosen such that we could keep it constant throughout all experiments (solid- and liquid-state). At the same time, to avoid signal distortions, we made sure that it remained smaller than the peak-to-peak line width in the CW EPR spectrum of free SL-R5 in solution. Spectral simulations were performed using the EasySpin software package^63^ and home-written scripts for MATLAB 2022a.

### LT MAS DNP

All DNP solid-state NMR spectra were acquired on a Bruker 9.4 T wide-bore magnet with an AVANCE-III-HD NMR console and a 3.2 mm DNP low-temperature double-resonance ^1^H/^13^C MAS probe. The spectrometer was equipped with a gyrotron for microwave irradiation of the sample. The field sweep coil of the NMR magnet was set to give microwave irradiation at the maximum DNP enhancement of TOTAPOL (263.334 GHz). The estimated power of the microwave beam at the output of the probe waveguide was 4 W. A thermocouple located 8.5 mm from the sample was used to measure the temperature of the NMR experiments. The sample temperature was ca. 103 K (in the presence of microwave irradiation) and the MAS frequency was 8 kHz. The radiofrequency (RF) field was 96 kHz for both the 90 and 180° ^1^H pulses. ^1^H recycle delays were determined using a saturation recovery scheme and were set to 1.3 × *T*_1_ (typically between 2 and 4 s). ^13^C-detected ^1^H NMR saturation recovery experiments were carried out under microwave irradiation as described in Pinon^59^ to determine the different time constants of the (exponential) recovery of the polarization. A train of 90° pulses (comprising 50 pulses separated by 1 ms) was used to saturate the ^1^H magnetization, with recovery of the ^1^H magnetization allowed during the polarization delay τ. For all cross-polarization (CP) experiments, the amplitude of the ^1^H RF field was ramped during the contact time to improve efficiency.

### SEM

Precipitates were centrifuged, separated, and washed three times with 1 mL of H_2_O. The pellet was resuspended in 1 mL of H_2_O and diluted 1:10 in H_2_O. A 10 μL aliquot of the resulting suspension was deposited onto a Thermanox coverslip and air-dried. The samples were sputter-coated with a gold layer under high vacuum using a Bal-Tec SCD 005 system. Imaging was performed using a Zeiss SEM Supra 55 VP at 20 kV, and particle radii in the aggregated samples were evaluated using ImageJ software.

## Supporting Information Available

EPR simulation parameters, relaxation data, rates and fits, reference measurements, and MS data (PDF)
Transparent Peer Review report available (PDF)

## References

[ref1] Nguyen T. L.; Cha B. G.; Choi Y.; Im J.; Kim J. Injectable dual-scale mesoporous silica cancer vaccine enabling efficient delivery of antigen/adjuvant-loaded nanoparticles to dendritic cells recruited in local macroporous scaffold. Biomaterials 2020, 239, 119859 10.1016/j.biomaterials.2020.119859. 32070828

[ref2] Juère E.; Caillard R.; Marko D.; Del Favero G.; Kleitz F. Smart protein-based formulation of dendritic mesoporous silica nanoparticles: toward oral delivery of insulin. Chemistr Eur. J. 2020, 26, 5195–5199. 10.1002/chem.202000773. PMC7217061 32057143

[ref3] Bialas F.; Reichinger D.; Becker C. F. W. Biomimetic and biopolymer-based enzyme encapsulation. Enzyme Microb Technol. 2021, 150, 109864 10.1016/j.enzmictec.2021.109864. 34489023

[ref4] von Baeckmann C.; Riva A.; Guggenberger P.; Kählig H.; Han S. W.; Inan D.; Del Favero G.; Berry D.; Kleitz F. Targeting gut bacteria using inulin-conjugated mesoporous silica nanoparticles. Adv. Mater. Interfaces 2022, 9, 2102558 10.1002/admi.202102558.

[ref5] Huang W.; Pan H.; Hu Z.; Wang M.; Wu L.; Zhang F. A functional bimodal mesoporous silica nanoparticle with redox/cellulase dual-responsive gatekeepers for controlled release of fungicide. Sci. Rep 2023, 13, 802 10.1038/s41598-023-27396-8. 36646732 PMC9842698

[ref6] Iriarte-Mesa C.; Jobst M.; Bergen J.; Kiss E.; Ryoo R.; Kim J. C.; Crudo F.; Marko D.; Kleitz F.; Del Favero G. Morphology-Dependent Interaction of Silica Nanoparticles with Intestinal Cells: Connecting Shape to Barrier Function. Nano Lett. 2023, 23, 7758–7766. 10.1021/acs.nanolett.3c00835. 37433061 PMC10450799

[ref7] Lechner C. C.; Becker C. F. A sequence-function analysis of the silica precipitating silaffin R5 peptide. J. Pept Sci. 2014, 20, 152–8. 10.1002/psc.2577. 25975421

[ref8] Senior L.; Crump M. P.; Williams C.; Booth P. J.; Mann S.; Perriman A. W.; Curnow P. Structure and function of the silicifying peptide R5. J. Mater. Chem. B 2015, 3, 2607–2614. 10.1039/C4TB01679C. 32262908

[ref9] Gascoigne L.; Magana J. R.; Atkins D. L.; Sproncken C. C. M.; Gumi-Audenis B.; Schoenmakers S. M. C.; Wakeham D.; Wanless E. J.; Voets I. K. Fractal-like R5 assembly promote the condensation of silicic acid into silica particles. J. Colloid Interface Sci. 2021, 598, 206–212. 10.1016/j.jcis.2021.04.030. 33905996

[ref10] Li Q.; Wang Y.; Zhang G.; Su R.; Qi W. Biomimetic mineralization based on self-assembling peptides. Chem. Soc. Rev. 2023, 52, 1549–1590. 10.1039/D2CS00725H. 36602188

[ref11] Kroger N.; Deutzmann R.; Sumper M. Polycationic peptides from diatom biosilica that direct silica nanosphere formation. Science 1999, 286, 1129–32. 10.1126/science.286.5442.1129. 10550045

[ref12] Heintze C.; Formanek P.; Pohl D.; Hauptstein J.; Rellinghaus B.; Kröger N. An intimate view into the silica deposition vesicles of diatoms. BMC Materials 2020, 2, 1–15. 10.1186/s42833-020-00017-8.

[ref13] Schneider A. F.; Kithil M.; Cardoso M. C.; Lehmann M.; Hackenberger C. P. Cellular uptake of large biomolecules enabled by cell-surface-reactive cell-penetrating peptide additives. Nat. Chem. 2021, 13, 530–539. 10.1038/s41557-021-00661-x. 33859390

[ref14] Han W.; MacEwan S. R.; Chilkoti A.; Lopez G. P. Bio-inspired synthesis of hybrid silica nanoparticles templated from elastin-like polypeptide micelles. Nanoscale 2015, 7, 12038–44. 10.1039/C5NR01407G. 26114664 PMC4499310

[ref15] Sprenger K. G.; Prakash A.; Drobny G.; Pfaendtner J. Investigating the role of phosphorylation in the binding of silaffin peptide R5 to silica with molecular dynamics simulations. Langmuir 2018, 34, 1199–1207. 10.1021/acs.langmuir.7b02868. 28981294

[ref16] Del Favero G.; Bialas F.; Grabher S.; Wittig A.; Brauer B.; Gerthsen D.; Echalier C.; Kamalov M.; Marko D.; Becker C. F. W. Silica particles with a quercetin-R5 peptide conjugate are taken up into HT-29 cells and translocate into the nucleus. Chem. Commun. (Camb) 2019, 55, 9649–9652. 10.1039/C9CC02215E. 31339160

[ref17] Luckarift H. R.; Spain J. C.; Naik R. R.; Stone M. O. Enzyme immobilization in a biomimetic silica support. Nat. Biotechnol. 2004, 22, 211–3. 10.1038/nbt931. 14716316

[ref18] Reichinger D.; Reithofer M.; Hohagen M.; Drinic M.; Tobias J.; Wiedermann U.; Kleitz F.; Jahn-Schmid B.; Becker C. F. W. A biomimetic, silaffin R5-based antigen delivery platform. Pharmaceutics 2023, 15, 121 10.3390/pharmaceutics15010121. PMC9866965 36678751

[ref19] Choi O.; Kim B.-C.; An J.-H.; Min K.; Kim Y. H.; Um Y.; Oh M.-K.; Sang B.-I. A biosensor based on the self-entrapment of glucose oxidase within biomimetic silica nanoparticles induced by a fusion enzyme. Enzyme Microb. Technol. 2011, 49, 441–445. 10.1016/j.enzmictec.2011.07.005. 22112615

[ref20] Kozak F.; Brandis D.; Pötzl C.; Epastoa L. M.; Reichingera D.; Polyanskyc A.; Zagrovicc B.; Daus F.; Geyer A.; Becker C. F.; Kurzbach D. An atomistic view on the mechanism of diatom peptide-guided biomimetic silica formation. Adv. Sci. 2024, 11, 2401239 10.1002/advs.202401239. PMC11321707 38874418

[ref21] Lechner C. C.; Becker C. F. Modified silaffin R5 peptides enable encapsulation and release of cargo molecules from biomimetic silica particles. Bioorg. Med. Chem. 2013, 21, 3533–41. 10.1016/j.bmc.2013.04.006. 23643899

[ref22] Strobl J.; Kozak F.; Kamalov M.; Reichinger D.; Kurzbach D.; Becker C. F. Understanding Self-Assembly of Silica-Precipitating Peptides to Control Silica Particle Morphology. Adv. Mater. 2023, 35, e2207586 10.1002/adma.202207586. 36509953 PMC11475327

[ref23] Lutz H.; Jaeger V.; Schmüser L.; Bonn M.; Pfaendtner J.; Weidner T. The structure of the diatom silaffin peptide R5 within freestanding two-dimensional biosilica sheets. Angew. Chem. Int. Ed 2017, 56, 8277–8280. 10.1002/anie.201702707. 28608998

[ref24] Ndao M.; Goobes G.; Emani P. S.; Drobny G. P. A REDOR ssNMR investigation of the role of an N-terminus lysine in R5 silica recognition. Langmuir 2018, 34, 8678–8684. 10.1021/acs.langmuir.5b04114. 27039990 PMC6785185

[ref25] Buckle E. L.; Roehrich A.; Vandermoon B.; Drobny G. P. Comparative study of secondary structure and interactions of the R5 peptide in silicon oxide and titanium oxide coprecipitates using solid-state NMR spectroscopy. Langmuir 2017, 33, 10517–10524. 10.1021/acs.langmuir.7b01048. 28898103 PMC6786483

[ref26] Roehrich A.; Ash J.; Zane A.; Masica D. L.; Gray J. J.; Goobes G.; Drobny G. Solid-state NMR studies of biomineralization peptides and proteins. Proteins at Interfaces III State of the Art 2012, 1120, 77–96. 10.1021/bk-2012-1120.ch004.

[ref27] Roeters S. J.; Mertig R.; Lutz H.; Roehrich A.; Drobny G.; Weidner T. Backbone structure of diatom silaffin peptide R5 in biosilica determined by combining solid-state nmr with theoretical sum-frequency generation spectra. J. Phys. Chem. Lett. 2021, 12, 9657–9661. 10.1021/acs.jpclett.1c02786. 34586816

[ref28] Ravera E.; Martelli T.; Geiger Y.; Fragai M.; Goobes G.; Luchinat C. Biosilica and bioinspired silica studied by solid-state NMR. Coord. Chem. Rev. 2016, 327, 110–122. 10.1016/j.ccr.2016.06.003.

[ref29] Kolbe F.; Daus F.; Geyer A.; Brunner E. Phosphate-silica interactions in diatom biosilica and synthetic composites studied by rotational echo double resonance (REDOR) NMR spectroscopy. Langmuir 2020, 36, 4332–4338. 10.1021/acs.langmuir.0c00336. 32233513

[ref30] Chen C. L.; Rosi N. L. Peptide-based methods for the preparation of nanostructured inorganic materials. Angew. Chem. Int. Ed 2010, 49, 1924–1942. 10.1002/anie.200903572. 20183835

[ref31] Marner W. D.; Shaikh A. S.; Muller S. J.; Keasling J. D. Enzyme immobilization via silaffin-mediated autoencapsulation in a biosilica support. Biotechnol. Prog. 2009, 25, 417–423. 10.1002/btpr.136. 19334285 PMC11245164

[ref32] Strobl J.; Kozak F.; Kamalov M.; Reichinger D.; Kurzbach D.; Becker C. F. Understanding self-assembly of silica-precipitating peptides to control silica particle morphology. Adv. Mater. 2023, 35, e2207586 10.1002/adma.202207586. 36509953 PMC11475327

[ref33] Felli I. C.; Brutscher B. Recent advances in solution NMR: fast methods and heteronuclear direct detection. ChemPhysChem 2009, 10, 1356–68. 10.1002/cphc.200900133. 19462391

[ref34] Felli I. C.; Pierattelli R. (13)C Direct detected NMR for challenging systems. Chem. Rev. 2022, 122, 9468–9496. 10.1021/acs.chemrev.1c00871. 35025504 PMC9136920

[ref35] Akbey U.; Oschkinat H. Structural biology applications of solid-state MAS DNP NMR. J. Magn. Reson. 2016, 269, 213–224. 10.1016/j.jmr.2016.04.003. 27095695

[ref36] Lesage A.; Lelli M.; Gajan D.; Caporini M. A.; Vitzthum V.; Mieville P.; Alauzun J.; Roussey A.; Thieuleux C.; Mehdi A.; Bodenhausen G.; Coperet C.; Emsley L. Surface enhanced NMR spectroscopy by dynamic nuclear polarization. J. Am. Chem. Soc. 2010, 132, 15459–61. 10.1021/ja104771z. 20831165

[ref37] Klug C. S.; Feix J. B. Methods and applications of site-directed spin labeling EPR spectroscopy. Methods in cell biology 2008, 84, 617–658. 10.1016/S0091-679X(07)84020-9. 17964945

[ref38] Ianiro A.; Wu H.; van Rijt M. M. J.; Vena M. P.; Keizer A. D. A.; Esteves A. C. C.; Tuinier R.; Friedrich H.; Sommerdijk N.; Patterson J. P. Liquid-liquid phase separation during amphiphilic self-assembly. Nat. Chem. 2019, 11, 320–328. 10.1038/s41557-019-0210-4. 30778139

[ref39] Polyansky A. A.; Gallego L. D.; Efremov R. G.; Kohler A.; Zagrovic B. Protein compactness and interaction valency define the architecture of a biomolecular condensate across scales. Elife 2023, 12, 1–23. 10.7554/eLife.80038. PMC10406433 37470705

[ref40] Toyama Y.; Rangadurai A. K.; Forman-Kay J. D.; Kay L. E. Mapping the per-residue surface electrostatic potential of CAPRIN1 along its phase-separation trajectory. Proc. Nat. Acad. Sci. 2022, 119, e2210492119 10.1073/pnas.2210492119. 36040869 PMC9457416

[ref41] Bermel W.; Bertini I.; Chill J.; Felli I. C.; Haba N.; Kumar M. V. V.; Pierattelli R. Exclusively heteronuclear (13) C-detected amino-acid-selective NMR experiments for the study of intrinsically disordered proteins (IDPs). ChemBioChem. 2012, 13, 2425–32. 10.1002/cbic.201200447. 23060071

[ref42] Murrali M. G.; Piai A.; Bermel W.; Felli I. C.; Pierattelli R. Proline dingerprint in intrinsically disordered proteins. ChemBioChem. 2018, 19, 1625–1629. 10.1002/cbic.201800172. 29790640

[ref43] Carper W. R.; Keller C. E. Direct determination of NMR correlation times from spin–lattice and spin–spin relaxation times. J. Phys. Chem. A 1997, 101, 3246–3250. 10.1021/jp963338h.

[ref44] Guseva S.; Schnapka V.; Adamski W.; Maurin D.; Ruigrok R. W. H.; Salvi N.; Blackledge M. Liquid-Liquid Phase Separation Modifies the Dynamic Properties of Intrinsically Disordered Proteins. J. Am. Chem. Soc. 2023, 145, 10548–10563. 10.1021/jacs.2c13647. 37146977 PMC10197138

[ref45] Unarta I. C.; Cao S.; Goonetilleke E. C.; Niu J.; Gellman S. H.; Huang X. Submillisecond atomistic molecular dynamics simulations reveal hydrogen bond-driven diffusion of a guest peptide in protein-RNA condensate. J. Phys. Chem. B 2024, 128, 2347–2359. 10.1021/acs.jpcb.3c08126. 38416758 PMC11057999

[ref46] Rauscher S.; Pomes R. The liquid structure of elastin. Elife 2017, 6, 1–21. 10.7554/eLife.26526. PMC5703643 29120326

[ref47] Karafiludis S.; Scoppola E.; Wolf S. E.; Kochovski Z.; Matzdorff D.; Van Driessche A. E.; Hövelmann J.; Emmerling F.; Stawski T. M. Evidence for liquid-liquid phase separation during the early stages of Mg-struvite formation. J. Chem. Phys. 2023, 159, 134503 10.1063/5.0166278. 37787132

[ref48] Murata K.; Tanaka H. Liquid-liquid transition without macroscopic phase separation in a water-glycerol mixture. Nat. Mater. 2012, 11, 436–43. 10.1038/nmat3271. 22426459

[ref49] Kurzbach D.; Schömer M.; Wilms V. S.; Frey H.; Hinderberger D. How structure-related collapse mechanisms determine nanoscale inhomogeneities in thermoresponsive polymers. Macromolecules 2012, 45, 7535–7548. 10.1021/ma3014299.

[ref50] Wertz J. Electron spin resonance: elementary theory and practical applications; Springer Science & Business Media: London, 2012.

[ref51] Bruno F.; Gigli L.; Ravera E. Spin label study of the orientational preferences of lysozyme in a bioinspired silica composite. J. Comp Sci. 2023, 7, 188 10.3390/jcs7050188.

[ref52] Schweiger A.; Jeschke G. Principles of pulse electron paramagnetic resonance; Oxford University Press: Oxford, U.K., 2001.

[ref53] Weil J. A.; Bolton J. R. Electron paramagnetic resonance: elementary theory and practical applications; John Wiley & Sons: 2007.

[ref54] Kurzbach D.; Junk M. J.; Hinderberger D. Nanoscale inhomogeneities in thermoresponsive polymers. Macromol. Rapid Commun. 2013, 34, 119–34. 10.1002/marc.201200617. 23169221

[ref55] Ravera E.; Michaelis V. K.; Ong T. C.; Keeler E. G.; Martelli T.; Fragai M.; Griffin R. G.; Luchinat C. Biosilic-entrapped enzymes studied by using dynamic nuclear polarization enhanced high-field NMR spectroscopy. ChemPhysChem 2015, 16, 2751–2754. 10.1002/cphc.201500549. 26266832 PMC4752418

[ref56] Geiger Y.; Gottlieb H. E.; Akbey U.; Oschkinat H.; Goobes G. Studying the conformation of a silaffin-derived pentalysine peptide embedded in bioinspired silica using solution and dynamic nuclear polarization magic-angle spinning NMR. J. Am. Chem. Soc. 2016, 138, 5561–7. 10.1021/jacs.5b07809. 26451953

[ref57] Venkatesh A.; Casano G.; Rao Y.; De Biasi F.; Perras F. A.; Kubicki D. J.; Siri D.; Abel S.; Karoui H.; Yulikov M. Deuterated TEKPol biradicals and the spin-diffusion barrier in MAS DNP. Angew. Chem. Int. Ed 2023, 135, e202304844 10.1002/ange.202304844. 37222433

[ref58] Perras F. A.; Wang L.-L.; Manzano J. S.; Chaudhary U.; Opembe N. N.; Johnson D. D.; Slowing I. I.; Pruski M. Optimal sample formulations for DNP SENS: The importance of radical-surface interactions. Curr. Opin. Colloid Interface Sci. 2018, 33, 9–18. 10.1016/j.cocis.2017.11.002.

[ref59] Pinon A. C.; Schlagnitweit J.; Berruyer P.; Rossini A. J.; Lelli M.; Socie E.; Tang M.; Pham T.; Lesage A.; Schantz S. Measuring nano-to microstructures from relayed dynamic nuclear polarization NMR. J. Phys. Chem. C 2017, 121, 15993–16005. 10.1021/acs.jpcc.7b04438.

[ref60] Hovav Y.; Feintuch A.; Vega S. Theoretical aspects of dynamic nuclear polarization in the solid state - The cross effect. J. Magn. Reson. 2012, 214, 29–41. 10.1016/j.jmr.2011.09.047. 22119645

[ref61] Wenckebach W. T. Spectral diffusion and dynamic nuclear polarization: Beyond the high temperature approximation. J. Magn. Reson. 2017, 284, 104–114. 10.1016/j.jmr.2017.10.001. 29028542

[ref62] Felli I. C.; Pierattelli R. Novel methods based on (13)C detection to study intrinsically disordered proteins. J. Magn. Reson. 2014, 241, 115–25. 10.1016/j.jmr.2013.10.020. 24656084

[ref63] Stoll S.; Schweiger A. EasySpin, a comprehensive software package for spectral simulation and analysis in EPR. J. Magn. Reson. 2006, 178, 42–55. 10.1016/j.jmr.2005.08.013. 16188474

