# Supplementary material for: R5 Peptides Constitute Condensed Phases with Liquid-Like Properties in Biomimetic Silica Capsules
Source: J Phys Chem Lett. 2025 Apr 23;16(17):4326–35. doi: 10.1021/acs.jpclett.5c00144 (PMC12051205; doi:10.1021/acs.jpclett.5c00144)
Supplement: Supplementary file 1 — jz5c00144_si_001.pdf [file jz5c00144_si_001.pdf]

Supporting Information

R5 peptides constitute condensed phases with liquid-like properties in biomimetic silica capsules

Dörte Brandis[1,2], Giulia Mollica[3], Dennis Kurzbach[1,]*

[1]Institute of Biological Chemistry, Faculty of Chemistry, University of Vienna, Währinger Str. 38, 1090 Vienna, Austria
[2]University of Vienna, Vienna Doctoral School in Chemistry (DoSChem), Währinger Str. 42, 1090 Vienna, Austria
[3] Aix Marseille Univ, CNRS, ICR, 13397 Marseille, France
**E-Mail: dennis.kurzbach@univie.ac.at*

Content

EPR Simulation Parameters (liquid)	Page S1
Relaxation data and fits of ^{13}C - detected R_1 measurements in H_2O.	Page S1
Relaxation data and fits of ^{13}C - detected R_2 measurements in H_2O.	Page S2
Relaxation fits of ^{13}C - detected R_1 measurements in 50 mM phosphate solution.	Page S2
Relaxation data and fits of ^{13}C - detected R_2 measurements in 50 mM phosphate solution.	Page S3
R_1 rates of ^{13}C – ^{15}N R5	Page S3
R_2 rates of ^{13}C – ^{15}N R5	Page S3
Liquid-state ^{13}C-^{15}N correlation spectrum of R5 encapsulated in silica shell	Page S4
CW EPR spectrum of the solution of the supernatant during wash cycles	Page S4
EPR Simulation Parameters (solid)	Page S5
Mass spectra of purified R5	Page S5

Table S1. Parameters used for simulation of the EPR spectrum of SL-R5 in H_2O and 50 mM phosphate.

Component	H_2O	50 mM P_i
Line width (peak-to-peak) / MHz	5.9	6.4
g_{iso} - value	2.0061	2.0058
A_{iso} MHz	45.03	46.00
τ_c / ns	0.04	0.37
Exchange frequency / ms^{-1}	0	2

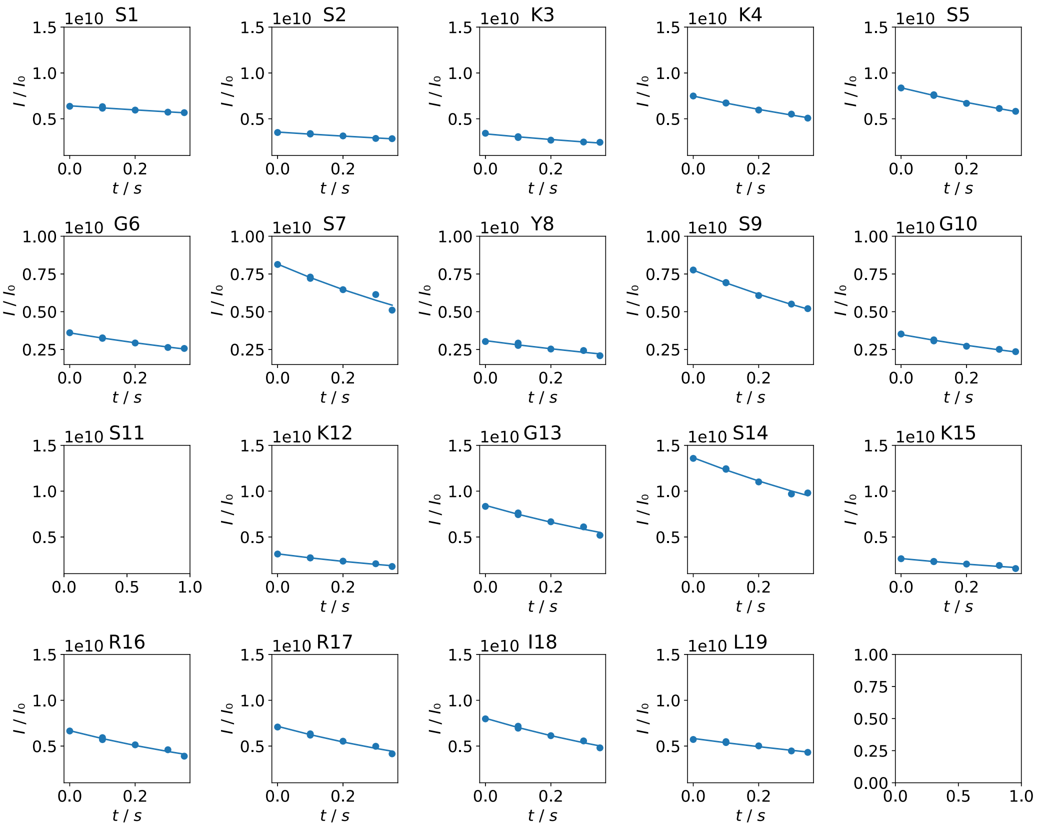

Figure S1. Relaxation data and fits of ^{13}C - detected R_1 measurements in H_2O.

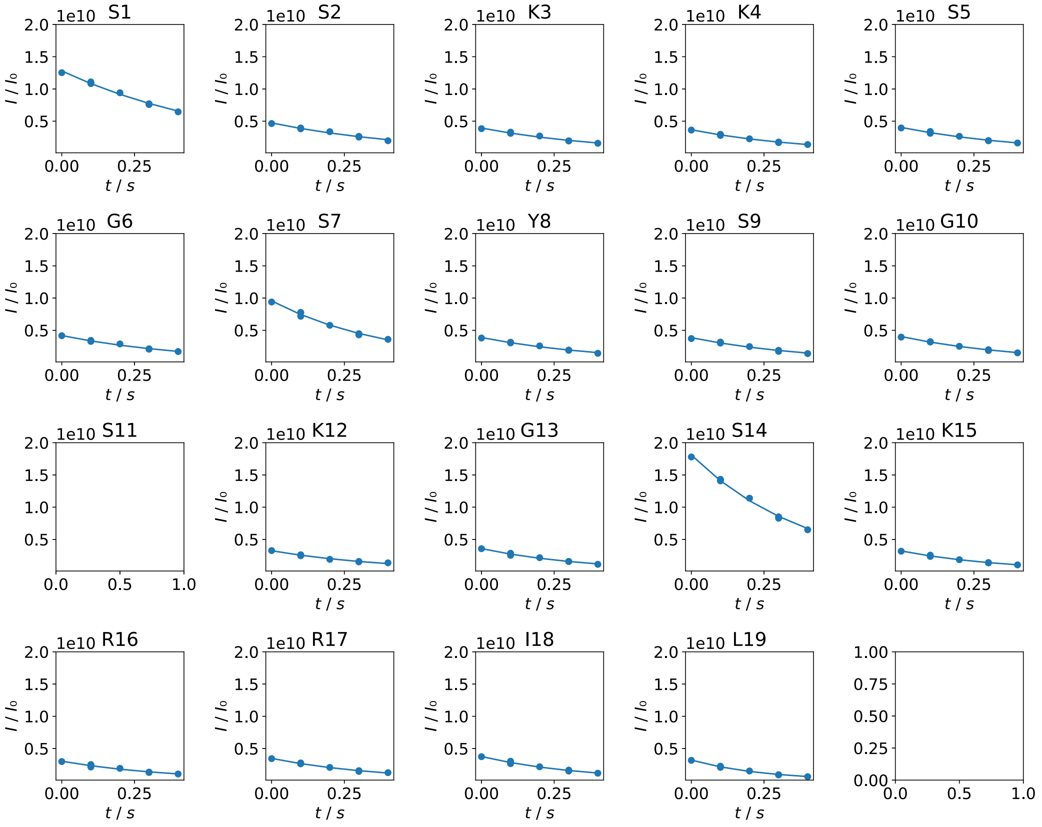

Figure S2. Relaxation data and fits of [13]C - detected R_2 measurements in H_2O.

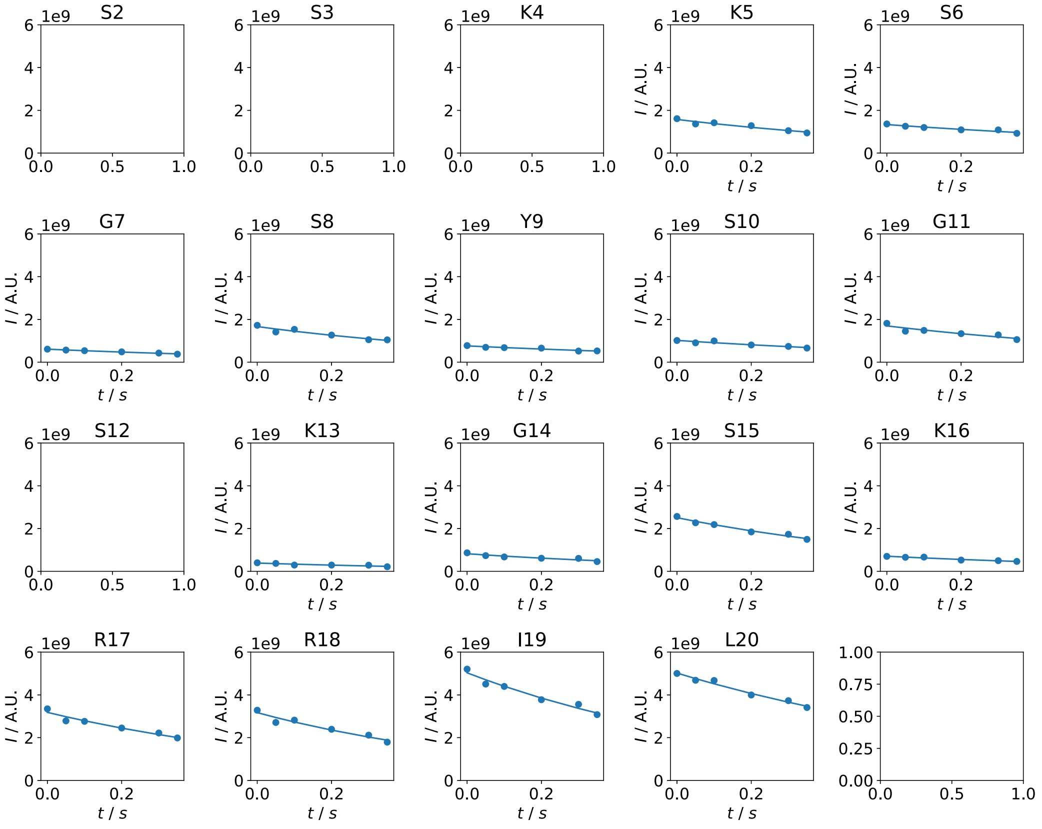

Figure S3. Relaxation fits of [13]C - detected R_1 measurements in 50 mM phosphate solution.

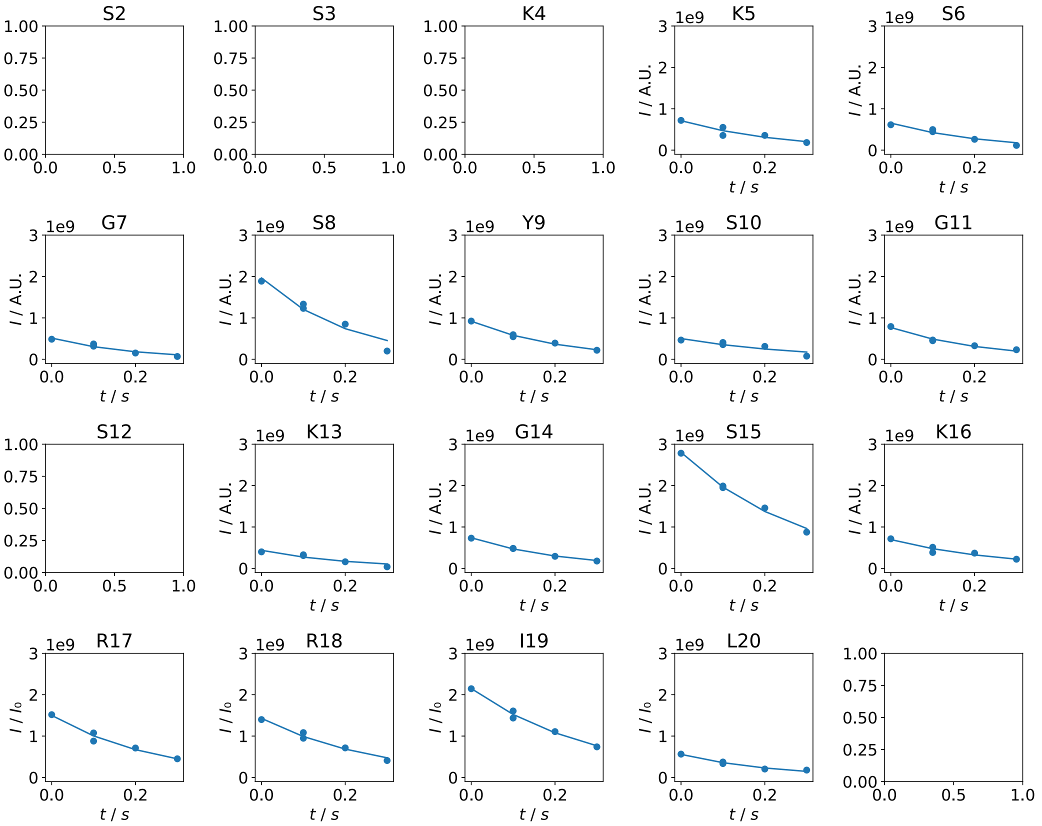

Figure S4. Relaxation data and fits of [13]C - detected R_2 measurements in 50 mM phosphate solution.

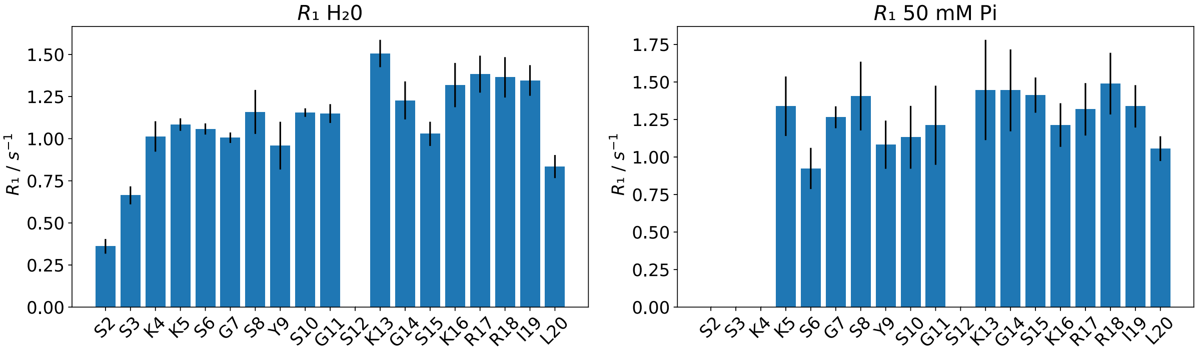

Figure S5. R_1 rates of [13]C – [15]N R5 in water (left) and 50 mM phosphate solution (right) measured with ^{13}C-detected relaxation measurements.

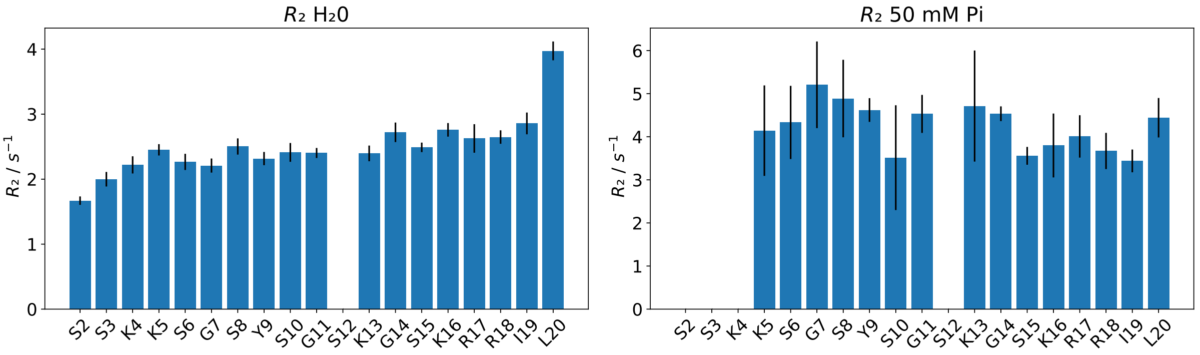

Figure S6. R_2 rates of [13]C – [15]N R5 in water (left) and 50 mM phosphate solution (right) measured with ^{13}C-detected relaxation measurements.

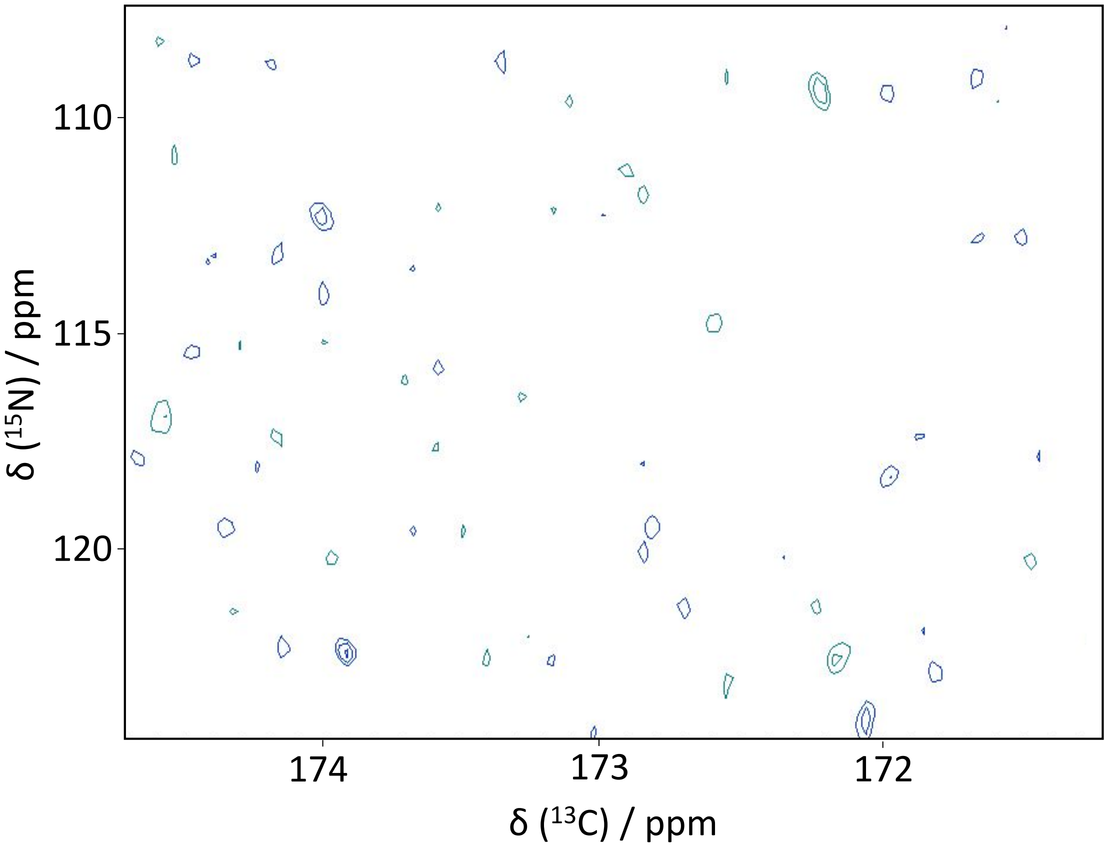

Figure S7. Liquid-state ^{13}C-^{15}N correlation spectrum of R5 encapsulated in silica shell. No signals from the peptide are visible.

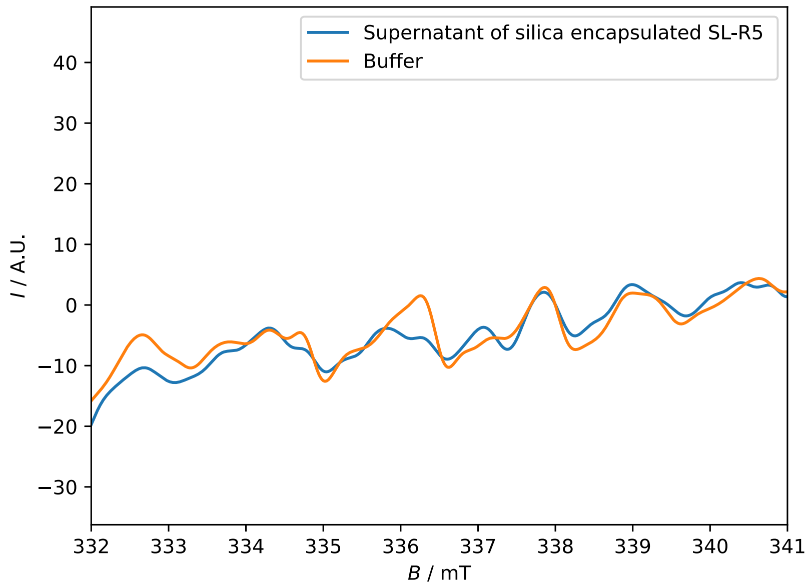

Figure S8. CW EPR spectrum of the solution of the supernatant during wash cycles (blue) compared with a CW EPR spectrum of the buffer, confirming that no peptide signal is detectable outside the particles.

Table S2. Parameters used for simulation of the EPR spectrum of SL-R5 containing silica particles. Tensors are given as principal components in the order [xx, yy, zz]. Note that using a slow-motion model to simulate the slow species with very long rotational correlation times (10-300 ns) did not improve the accuracy of the spectral simulations.

**Component**	**fast**	**slow**	**additional**
Voigt line width (Lorentzain + Gaussian) / mT	[0.03 0]	[0.49 0]	[0.28 0.27]
g - tensor	[2.0087, 2.0081, 2.0036]	[2.0087, 2.0081, 2.0036]	[2.0087, 2.0081, 2.0036]
A_{iso} / A – tensor / MHz	42.3	[18, 18, 104]	[18, 18, 109]
$Log_{10}(D_{rot})$ / rad^2ns^{-1}	[7.5, 9.0, 8.0]	n/a	[8.0, 8.0, 8.5]
Exchange frequency / ms^{-1}	40	0	10
Weight fraction	0.4	0.5	0.1

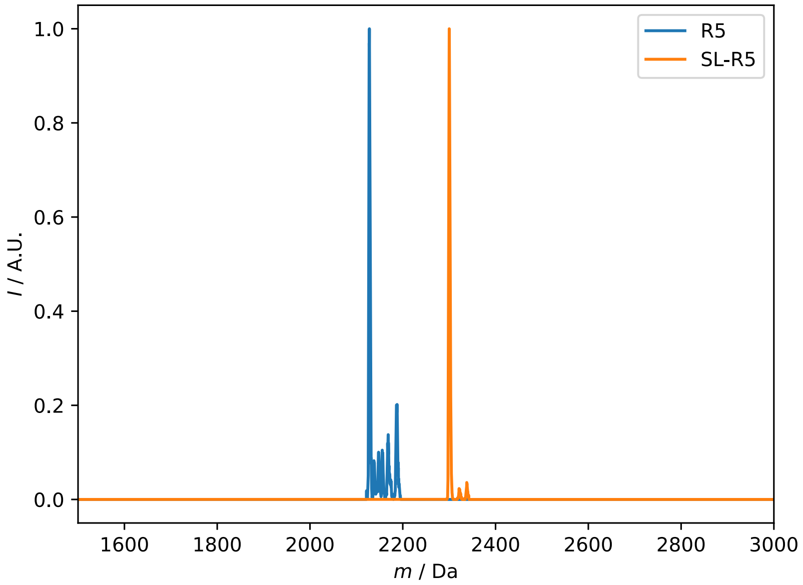

Figure S9. Mass spectra of purified R5 before (blue) and after (orange) labeling with MTSL confirming successful labeling reaction.
