# Supplementary material for: R5 Peptides Constitute Condensed Phases with Liquid-Like Properties in Biomimetic Silica Capsules
Source: J Phys Chem Lett. 2025 Apr 23;16(17):4326–35. doi: 10.1021/acs.jpclett.5c00144 (PMC12051205; doi:10.1021/acs.jpclett.5c00144)
Supplement: Supplementary file 2 — jz5c00144_si_003.pdf [file jz5c00144_si_003.pdf]

jz-2025-00144d.R1

Name: Peer Review Information for "R5 peptides constitute condensed phases with liquid-like properties in biomimetic silica capsules"

First Round of Reviewer Comments

Reviewer: 1

Comments to the Author

1. Major advance reported in the paper:

The dynamic, liquid-like nature of the biotechnologically crucial silaffin-derivative R5 is a central feature for host-guest interactions in drug delivery applications and the use of encapsulated enzymes and nanoreactors. Therefore, a detailed analysis of the heterogeneous phases of R5 is pivotal for a deeper understanding of the properties of such materials and can foster new ideas about the tuning of such properties for specific applications.

2. Immediate significance of this advance

Different dynamics of the peptides in the silica were found using an elegant DNP/NMR/EPR approach. The presence of heterogeneous populations of R5 discovered in this work increases the understanding of the properties of such materials.

3. Technical suggestions (issues to be addressed before publication):

- The rotational correlation times obtained from the simulation of the spectra in Fig. 2 could be written in the inset or in the legend (40 ps, 370 ps, isotropic motion). It would be informative to know the concentration of the peptide used. In supporting Table 1 the g tensor parameters are provided. Were they taken from literature (citation needed)? Otherwise only giso can be obtained with accuracy from the fast motion X-band spectra (as Aiso) if the B field was calibrated (missing info on the calibration of the B field).

- Why using 2G mod amplitude for the spectra? With such fast dynamics, the linewidth might have been disturbed. Can the authors comment on that?

- The presence of a very mobile and a very immobile component is evident from the spectra in Fig. 4. However, the existence of three distinct populations (the third with only 10% weight) is not fully supported by the data analysis due to the complex anisotropic motions which are difficult to simulate without a multifrequency analysis and in the presence of exchange coupling.

- Labeling efficiency from mass spec data should be quantified (100%?). To simplify data analysis and validate the conclusion drawn, the spectra presented could be measured also on spin diluted EPR samples. By adding the spin-labeled proteins in a 1:10, or 1:20 ratio to the unlabeled variants and repeating the experiments shown in Fig. 2 and 4, the exchange will be minimized, and the rotational dynamics might be described more accurately. In Fig. 2 the effect of exchange on the overall linewidth can be verified, and in Fig. 4 the two- or three- component analysis could be better compared.

- The immobile component in Fig. 4 is simulated with a spectrum in the rigid limit (without exchange), however, X-band EPR is sensitive up to about 30-50 ns rotational correlation times (to improve clarity, the sensitive range of X-band cw EPR could be explained in the introduction). Such slow rotational correlation time could aid the spectral simulation instead of a powder spectrum?

- The bi-exponential nature of the DNP build-up curves points towards the presence of at least two distinct R5 phases, but three phases are suggested by EPR. Can we say that the phases responsible for DNP should only be the liquid-like ones? In that case, the biexponential amplitudes should reflect the 4:1 ratio of the two EPR liquid-like components. Could slow rotational correlation times in the order of 30 ns be still useful for efficient DNP? Can we rule out that the more immobile phase does not contribute to the build-up curves? The authors could comment on that.

- it is not clear why the authors can describe the dynamic phase as 'condensed liquid phase' . Is it possible to distinguish a highly concentrated protein solution from a condensate (a sin liquid-liquid phase separated systems) in this material? The authors can further comment on this point.

Reviewer: 2

Comments to the Author

This paper is relevant contribution to the field. However, I advise the authors to provide a clearer and substantiated explanation about the usage of biomimetic silica capsules when compared to other traditional methods. Furthermore, it would benefit the paper if the authors could make a statement about the application of these capsules to design of bio-functional hybrid nanomaterials.

Author's Response to Peer Review Comments:

To
Phys. Chem. Lett.
Editorial Office

Assoc. Prof. Dr. Dennis Kurzbach
University Vienna
Faculty of Chemistry
Institute of Biological Chemistry
Währinger Straße 38
1090 Vienna
Austria
Mail.: Dennis.Kurzbach@univie.ac.at
Tel.: +43-1-4277-70528

Vienna, 11/03/2025

Dear Editor,

we enclose a revised electronic version of the manuscript by Brandis *et al.*, entitled

R5 peptides constitute condensed phases with liquid-like properties in biomimetic silica capsules

that we would like to submit for re-consideration for the Journal of Physical Chemistry Letters.

We have responded to all comments of the referees in a point-by-point discussion (please find below the referee comments in black and our answers in green) and revised the manuscript according to the editorial guidelines and referees' comments. We highlighted all scientific revisions in the manuscript in yellow and editorial changes in green.

Sincerely and on behalf of all authors,

Dennis Kurzbach

Reviewer: 1

Recommendation: This paper may be publishable, but major revision is needed; I would like to be invited to review any future revision.

Comments:

1. Major advance reported in the paper:

The dynamic, liquid-like nature of the biotechnologically crucial silaffin-derivative R5 is a central feature for host-guest interactions in drug delivery applications and the use of encapsulated enzymes and nanoreactors. Therefore, a detailed analysis of the heterogeneous phases of R5 is pivotal for a deeper understanding of the properties of such materials and can foster new ideas about the tuning of such properties for specific applications.

We thank the referee for his motivating feedback.

2. Immediate significance of this advance Different dynamics of the peptides in the silica were found using an elegant DNP/NMR/EPR approach. The presence of heterogeneous populations of R5 discovered in this work increases the understanding of the properties of such materials.

Again, we thank the referee.

3. Technical suggestions (issues to be addressed before publication):

- The rotational correlation times obtained from the simulation of the spectra in Fig. 2 could be written in the inset or in the legend (40 ps, 370 ps, isotropic motion). It would be informative to know the concentration of the peptide used. In supporting Table 1 the g tensor parameters are provided. Were they

taken from literature (citation needed)? Otherwise only giso can be obtained with accuracy from the fast motion X-band spectra (as Aiso) if the B field was calibrated (missing info on the calibration of the B field).

We followed the referee's advice and added the rotational correlation times to the figure captions. We also thank the referee for catching that the peptide concentrations were not explicitly reported for all experiments (2 mg/mL). We have added this information now to the manuscript. Furthermore, we have also followed his correct suggestion to report only isotropic values (the tensor reported was used in the simulation code but is indeed averaged at X-band and ambient temperatures). The B-field was calibrated, too. We added this information as well.

- Why using 2G mod amplitude for the spectra? With such fast dynamics, the linewidth might have been disturbed. Can the authors comment on that?

2 G was used as a value that worked for both the liquid and the solid-state spectra. The modulation amplitude remained below the peak-to-peak linewidth in the narrowest spectrum (i.e., free R5 in solution) to avoid line shape perturbations. Following the referee's advice we have added an explanatory comment to the revised manuscript.

> "The modulation amplitude was chosen such that we could keep it constant throughout all experiments (solid- and liquid-state). At the same time, to avoid signal distortions, we made sure that it remained smaller than the peak-to-peak linewidth in the CW EPR spectrum of free SL-R5 in solution."

- The presence of a very mobile and a very immobile component is evident from the spectra in Fig. 4. However, the existence of three distinct populations (the third with only 10% weight) is not fully supported by the data analysis due to the complex anisotropic motions which are difficult to simulate without a multifrequency analysis and in the presence of exchange coupling.

We thank the referee for pointing this out. Indeed, it is challenging (if not impossible) to quantify exchange frequencies, linewidths etc. from a single spectrum. However, our data interpretation does not rely on quantification, but on (qualitative) observation of at least two different species with liquid-like and solid-like dynamics. We now realized that this point was only very briefly commented on in the original manuscript. To take the referee's correct point into account, we have therefore added the following paragraph to the manuscript, which stresses that the reported parameters are the result of our data fitting routine, but have to be carefully interpreted as one of possible many solutions (which yet all lead to the presence of three species):

> "The reported exchange frequencies and relative populations are the values fed into our spectral simulations to match the experimental spectra. However, given the complexity of EPR spectra in the slow-motion regime, including anisotropic rotational diffusion, it cannot be ruled out that other combinations of parameters would lead to similar results. Therefore, it is important to note that our data interpretation does not rely on quantification of exchange frequencies of relative populations, but on the mere observation that only two components (fast and slow rotating species) could not provide a good match to the experimental data."

- Labeling efficiency from mass spec data should be quantified (100%?). To simplify data analysis and validate the conclusion drawn, the spectra presented could be measured also on spin diluted EPR samples. By adding the spin-labeled proteins in a 1:10, or 1:20 ratio to the unlabeled variants and repeating the experiments shown in Fig. 2 and 4, the exchange will be minimized, and the rotational dynamics might be described more accurately. In Fig. 2 the effect of exchange on the overall linewidth can be verified, and in Fig. 4 the two- or three- component analysis could be better compared.

We have added the determined labelling efficiency, which was in fact 100 %. We thank the referee for suggesting that the EPR experiments be repeated via titration experiments at different labelling degrees. However, such a study would require lengthy new experiments (including sample reproduction) to quantitatively deconvolute the linewidth and exchange contributions, which is not necessary to confirm our data interpretation. The findings of our manuscript, i.e., the identification of liquid-like dynamics within the R5 capsules, are already fully supported by the presented results. Hence, the scientific outcome would remain similar. Overall, although we do agree with the reviewer that the suggested experiments would enable a more quantitative characterization of the dynamics present in the studied systems, these experiments should be the object of a dedicated study and, hence, remain out of the scope of the present investigation.

- The immobile component in Fig. 4 is simulated with a spectrum in the rigid limit (without exchange), however, X-band EPR is sensitive up to about 30-50 ns rotational correlation times (to improve clarity, the sensitive range of X-band cw EPR could be explained in the introduction). Such slow rotational correlation time could aid the spectral simulation instead of a powder spectrum?

Following the referee's advice, we have added a short discussion to the limits of X-band EPR to the manuscript. It reads:

> "X-band EPR spectroscopy is sensitive to molecular dynamics with rotational correlation times ranging from 10 ps to ca. 50 ns,[1-2] capturing motion on timescales from fast tumbling to near-rigid behavior. In terms of concentration, CW-EPR can detect radicals down to 10^{-9} M. At cryogenic temperatures (~4 K), sensitivity improves, allowing detection limits as low as 10^{-10} M under ideal conditions and especially in systems with long T_1 times.[1-2] In comparison, higher-frequency EPR techniques (e.g., Q-band at 35 GHz or W-band at 95 GHz) provide enhanced resolution but often demand higher sample concentrations."

Similarly, we have re-performed the simulation with a slow-tumbling model as suggested; however, the results did not improve (Fig. R1). This point further aligns with our above response that two components alone do not suffice to match the experimental EPR line shape. We have added this information to the revised manuscript.

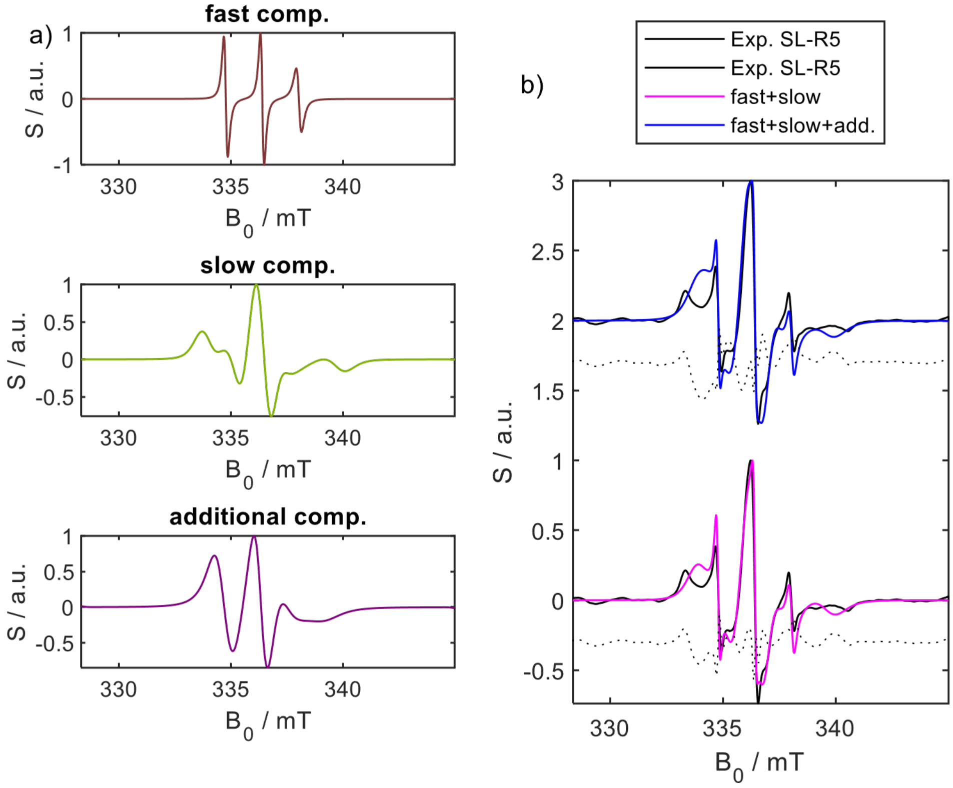

Figure R1. Reproduction of Fig. 4 of the manuscript with a slow-motion model ("chili" routine in Easy Spin) used to simulate the slow component (green). Here with a τ_c of 10 ns. The results did not improve. This was also observed for other probed values between 300 and 10 ns.

- The bi-exponential nature of the DNP build-up curves points towards the presence of at least two distinct R5 phases, but three phases are suggested by EPR. Can we say that the phases responsible for DNP should only be the liquid-like ones? In that case, the biexponential amplitudes should reflect the 4:1 ratio of the two EPR liquid-like components. Could slow rotational correlation times in the order of 30 ns be still useful for efficient DNP? Can we rule out that the more immobile phase does not contribute to the build-up curves? The authors could comment on that.

We thank the referee for pointing out this possibility. We have added a comment to the manuscript. However, we want to stress that DNP amplitudes depend on various factors, from local radical density to proton concentrations, such that the quantitative comparison with the liquid-state experiments cannot be undertaken. Similarly, (and apologies if we overlook something obvious here) the MAS DNP experiments are performed in vitrified solutions such that rotational correlations do not apply. To avoid any further confusion, we now added another paragraph to the revised manuscript explaining the relation between DNP and EPR data in more detail:

> "Finally, it should be noted that rotational diffusion plays no role in the interpretation of the MAS DNP spectra, as these were carried out at a temperature of 100 K. Therefore, a direct comparison of the EPR and the DNP data in terms of relative contributions of the different phases is not possible in a straightforward manner. However, the presence of two well distinct build-up time components strongly

supports the presence of at least two (vitrified) R5 phases with different radical concentrations, hence, agreeing with the identification of multiple dynamic components by EPR."

- it is not clear why the authors can describe the dynamic phase as 'condensed liquid phase' . Is it possible to distinguish a highly concentrated protein solution from a condensate (a sin liquidliquid phase separated systems) in this material? The authors can further comment on this point.

We now realized that our initial comment on this point was only very brief (we only referred to "condensed phases with liquid-like internal dynamics"). We have now added the following paragraph to the manuscript to make clear that we do not want to define the actual phase behavior with this notion, but instead determine liquid-like dynamics in terms of mobility. The added paragraph reads:

> "We chose the notion of "condensed phases with liquid-like dynamics" not to imply any relations to liquid-liquid phase separation[3-4], peptide condensation[5-6] or similar phenomena[7] that recently received ample attention and are subject to particular definitions in terms of phase behavior. Instead, we simply want to refer to a liquid-like character in terms of molecular dynamics of peptide-rich R5 phases while not implying any specific definition."

Reviewer: 2

Recommendation: This paper is publishable subject to minor revisions noted. Further review is not needed.

Comments:
This paper is relevant contribution to the field. However, I advise the authors to provide a clearer and substantiated explanation about the usage of biomimetic silica capsules when compared to other traditional methods. Furthermore, it would benefit the paper if the authors could make a statement about the application of these capsules to design of bio-functional hybrid nanomaterials.

We have added such a paragraph, it reads:

> "Traditional silica formation methods, such as sol-gel chemistry, often require extreme pH conditions, organic solvents, or high temperatures, which can compromise the stability and functionality of biological molecules. In contrast, nature-inspired approaches utilizing peptides, such as the R5 peptide enables the controlled deposition of silica under mild, aqueous conditions at neutral pH.[8-12] It facilitates silica formation by promoting biomimetic mineralization, leading to tunable porosity, enhanced structural stability, and the preservation of biocompatibility.[8, 10, 13] This method has opened new possibilities in diverse applications, including enzyme immobilization for biocatalysis, drug delivery systems for controlled release, and biosensors for diagnostic purposes.[13-14]"

References

1. Schweiger, A.; Jeschke, G., *Principles of pulse electron paramagnetic resonance*. Oxford university press: 2001.
2. Weil, J. A.; Bolton, J. R., *Electron paramagnetic resonance: elementary theory and practical applications*. John Wiley & Sons: 2007.
3. Ianiro, A.; Wu, H.; van Rijt, M. M. J.; Vena, M. P.; Keizer, A. D. A.; Esteves, A. C. C.; Tuinier, R.; Friedrich, H.; Sommerdijk, N.; Patterson, J. P., Liquid-liquid phase separation during amphiphilic self-assembly. *Nat Chem* **2019,** *11* (4), 320-328. 10.1038/s41557-019-0210-4
4. Murata, K.; Tanaka, H., Liquid-liquid transition without macroscopic phase separation in a water-glycerol mixture. *Nat Mater* **2012,** *11* (5), 436-43. 10.1038/nmat3271
5. Strobl, J.; Kozak, F.; Kamalov, M.; Reichinger, D.; Kurzbach, D.; Becker, C. F., Understanding Self-Assembly of Silica-Precipitating Peptides to Control Silica Particle Morphology. *Adv Mater* **2022**, e2207586. 10.1002/adma.202207586
6. Kozak, F.; Brandis, D.; Pötzl, C.; Epastoa, L. M.; Reichingera, D.; Polyanskyc, A.; Zagrovicc, B.; Daus, F.; Geyer, A.; Becker, C. F.; Kurzbach, D., An atomistic view on the mechanism of diatom peptide-guided biomimetic silica formation. *Adv. Sci.* **2024**, 2401239.
7. Kurzbach, D.; Schömer, M.; Wilms, V. S.; Frey, H.; Hinderberger, D., How structurerelated collapse mechanisms determine nanoscale inhomogeneities in thermoresponsive polymers. *Macromolecules* **2012,** *45* (18), 7535-7548.
8. Lechner, C. C.; Becker, C. F., Modified silaffin R5 peptides enable encapsulation and release of cargo molecules from biomimetic silica particles. *Bioorg Med Chem* **2013,** *21* (12), 3533-41. 10.1016/j.bmc.2013.04.006
9. Lechner, C. C.; Becker, C. F., A sequence-function analysis of the silica precipitating silaffin R5 peptide. *J Pept Sci* **2014,** *20* (2), 152-8. 10.1002/psc.2577
10. Senior, L.; Crump, M. P.; Williams, C.; Booth, P. J.; Mann, S.; Perriman, A. W.; Curnow, P., Structure and function of the silicifying peptide R5. *J Mater Chem B* **2015,** *3* (13), 2607-2614. 10.1039/c4tb01679c
11. Del Favero, G.; Bialas, F.; Grabher, S.; Wittig, A.; Brauer, B.; Gerthsen, D.; Echalier, C.; Kamalov, M.; Marko, D.; Becker, C. F. W., Silica particles with a quercetin-R5 peptide conjugate are taken up into HT-29 cells and translocate into the nucleus. *Chem Commun (Camb)* **2019,** *55* (65), 9649-9652. 10.1039/c9cc02215e
12. Gascoigne, L.; Magana, J. R.; Atkins, D. L.; Sproncken, C. C. M.; Gumi-Audenis, B.; Schoenmakers, S. M. C.; Wakeham, D.; Wanless, E. J.; Voets, I. K., Fractal-like R5 assembly promote the condensation of silicic acid into silica particles. *J Colloid Interface Sci* **2021,** *598*, 206-212. 10.1016/j.jcis.2021.04.030
13. Han, W.; MacEwan, S. R.; Chilkoti, A.; Lopez, G. P., Bio-inspired synthesis of hybrid silica nanoparticles templated from elastin-like polypeptide micelles. *Nanoscale* **2015,** *7* (28), 12038-44. 10.1039/c5nr01407g
14. Zhang, Y. N.; Avery, R. K.; Vallmajo-Martin, Q.; Assmann, A.; Vegh, A.; Memic, A.; Olsen, B. D.; Annabi, N.; Khademhosseini, A., A Highly Elastic and Rapidly Crosslinkable Elastin-Like Polypeptide-Based Hydrogel for Biomedical

Applications. *Adv Funct Mater* **2015,** *25* (30), 4814-4826. 10.1002/adfm.201501489

jz-2025-00144d.R2

Name: Peer Review Information for "R5 peptides constitute condensed phases with liquid-like properties in biomimetic silica capsules"

Second Round of Reviewer Comments

Reviewer: 1

Comments to the Author

The authors carefully addressed all issues, providing supplementary information and adding detailed explanations in the text that overall improved the clarity of the presentation and strengthen the data interpretation. The manuscript should be published as it is.

Author's Response to Peer Review Comments:

To
Phys. Chem. Lett.
Editorial Office

Assoc. Prof. Dr. Dennis Kurzbach
University Vienna
Faculty of Chemistry
Institute of Biological Chemistry
Währinger Straße 38
1090 Vienna
Austria
Mail.: Dennis.Kurzbach@univie.ac.at

Tel.: +43-1-4277-70528

Vienna, 09/04/2025

Dear Editor,

we enclose a newly revised electronic version of the manuscript by Brandis *et al.*, entitled

R5 peptides constitute condensed phases with liquid-like properties in biomimetic silica capsules

that we would like to submit for re-consideration for the Journal of Physical Chemistry Letters.

We removed all section headings from the text body, keeping only Methods, Abstract, and TOC Graphic headings. We hope that you'll find the manuscript now suitable for publication.

Sincerely and on behalf of all authors,

Dennis Kurzbach
